# Carotid and Femoral Atherosclerotic Plaques in Asymptomatic and Non-Treated Subjects: Cardiovascular Risk Factors, 10-Years Risk Scores, and Lipid Ratios’ Capability to Detect Plaque Presence, Burden, Fibro-Lipid Composition and Geometry

**DOI:** 10.3390/jcdd7010011

**Published:** 2020-03-19

**Authors:** Mariana Marin, Daniel Bia, Yanina Zócalo

**Affiliations:** Departamento de Fisiología, Facultad de Medicina, Centro Universitario de Investigación, Innovación y Diagnóstico Arterial (CUiiDARTE), Universidad de la República, Montevideo 11800, Uruguay; cuiidarte@fmed.edu.uy (M.M.); dbia@fmed.edu.uy (D.B.)

**Keywords:** aortic pressure, asymptomatic subjects, atherosclerotic plaques, cardiovascular risk, carotid, femoral, lipid ratios, plaque composition, subclinical atherosclerosis, virtual histology

## Abstract

Carotid and/or femoral atherosclerotic plaques (AP) assessment through imaging studies is an interesting strategy for improving individual cardiovascular risk (CVR) stratification and cardiovascular disease (CVD) and/or events prediction. There is no consensus on who would benefit from image screening aimed at determining AP presence, burden, and characteristics. Aims: (1) to identify, in asymptomatic and non-treated subjects, demographic factors, anthropometric characteristics and cardiovascular risk factors (CRFs), individually or grouped (e.g., CVR equations, pro-atherogenic lipid ratios) associated with carotid and femoral AP presence, burden, geometry, and fibro-lipid content; (2) to identify cut-off values to be used when considering the variables as indicators of increased probability of AP presence, elevated atherosclerotic burden, and/or lipid content, in a selection scheme for subsequent image screening. Methods: CRFs exposure and clinical data were obtained (*n* = 581; *n* = 144 with AP; 47% females). Arterial (e.g., ultrasonography) and hemodynamic (central [cBP] and peripheral blood pressure; oscillometry/applanation tonometry) data were obtained. Carotid and femoral AP presence, burden (e.g., AP number, involved territories), geometric (area, width, height) and fibro-lipid content (semi-automatic, virtual histology analysis, grayscale analysis and color mapping) were assessed. Lipid profile was obtained. Lipid ratios (Total cholesterol/HDL-cholesterol, LDL-cholesterol/HDL-cholesterol, LogTryglicerides(TG)/HDL-cholesterol) and eight 10-years [y.]/CVR scores were quantified (e.g., Framingham Risk Scores [FRS] for CVD). Results: Age, 10-y./CVR and cBP showed the highest levels of association with AP presence and burden. Individually, classical CRFs and lipid ratios showed almost no association with AP presence. 10-y./CVR levels, age and cBP enabled detecting AP with large surfaces (˃p75th). Lipid ratios showed the largest association with AP fibro-lipid content. Ultrasound evaluation could be considered in asymptomatic and non-treated subjects aiming at population screening of AP (e.g., ˃ 45 y.; 10-y./FRS-CVD ˃ 5–8%); identifying subjects with high atherosclerotic burden (e.g., ˃50 y., 10-y./FRS-CVD ˃ 13–15%) and/or with plaques with high lipid content (e.g., LogTG/HDL ˃ 0.135).

## 1. Introduction

Early detection and treatment of atherosclerotic disease might be central for improving cardiovascular (CV) prevention [1]. Plaques assessment with vascular imaging is an appealing strategy to aid in CV events’ prediction and a potential tool for improving individual CV risk (CVR) stratification, enabling more efficient prevention [1,2,3,4,5]. Atherosclerotic load or burden (i.e., number of plaques and/or territories affected), is associated with (gradual) increase in CVR [3,6,7]. In turn, plaque geometry (e.g., volume, area) and composition (e.g., lipid content, intraplaque hemorrhage) are determinants of plaque vulnerability (i.e., risk of instability and plaque accident) [8,9,10]. Subjects with vulnerable plaques would have increased risk of CV events [11]. Then, determining plaques presence, atherosclerotic burden and/or quantifying vulnerability based on the geometry and/or composition of the plaque(s) would be of value when planning and implementing specific preventive strategies [9,12,13,14,15].

CV risk factors (CRFs) as such, have shown independent associations with atherosclerosis, which would differ depending on the factor, subject, and arterial pathway considered. On the other hand, the 10-year global CVR (10-y./CVR) assessed in asymptomatic individuals through recommended risk equations (e.g., Framingham Risk Score [FRS]) could be modified and the risk could be reclassified if atherosclerotic plaques were detected in carotid or femoral arteries [1,3,4,5,6,7,9]. Therefore, the use of non-invasive imaging techniques to improve CVR assessment has raised ongoing interest. In people with 10-y/CVR close to the decision threshold imaging techniques could be considered to improve risk prediction and decision making [5,9].

So far there is no consensus on who would benefit from routine ultrasound evaluation of the carotid and femoral arteries to determine presence, burden, and characteristics of atherosclerotic plaques to assess CV risk on an individual basis.

In this context, it should be noted that it is unknown the extent to which CRFs, risk equations (e.g., FRS, ASSIGN Risk Score) and/or risk markers (e.g., pro-atherogenic lipid ratios) are related to the presence, burden, geometry and composition of carotid and/or femoral atherosclerotic plaques. It is also unknown which cut-off levels should be considered for the South American population when using CRFs, risk equations and/or risk markers to accurately identify subjects most likely to present subclinical atherosclerosis who would particularly benefit from further evaluation (e.g., imaging studies).

This work aimed at: (1) identifying in asymptomatic and non-treated subjects, demographic factors, anthropometric characteristics, and/or CRFs, considered “separately” or “grouped” (e.g., in CVR equations or lipid ratios) associated with (a) plaque presence, (b) atherosclerotic burden and (c) geometry and composition of carotid and femoral atherosclerotic plaques; (2) determining the cut-off values to be considered for the analyzed variables (i.e., demographic, anthropometric, CRFs, 10-y./CVR equations and lipid ratios) to identify subjects with increased likelihood of carotid and femoral plaques presence, major atherosclerotic burden and/or plaques vulnerability, in order to set selection-schema for subsequent image screening. As an additional aim, we identified and compared the levels of association between the geometry and composition of the plaques.

## 2. Materials and Methods

### 2.1. Study Population

The study was carried out in the context of the Centro Universitario de Investigación, Innovación y Diagnóstico Arterial (CUiiDARTE) Project [16,17,18,19]. We considered data from a total of 581 subjects (47% females) provided by CUiiDARTE Database. This includes demographic, anthropometric, clinical data, and information related with CRFs exposure and structural and functional arterial parameters non-invasively obtained in community-based projects. Subjects included in this work were ˃18 y. of age and met the following criteria: all were asymptomatic and in stable clinical conditions, none of them had congenital, chronic or infectious diseases, previous history of CV disease (CVD) or events (myocardial infarction [MI], angina, heart failure, stroke, transient ischemic attack, aortic disease, coronary heart disease [CHD] and peripheral artery disease), none of them were on drug treatment (i.e., lipid lowering—statins or fibrates—antihypertensive and/or hypoglycemic) (Table 1). Exclusion criteria included rhythm other than sinus rhythm and valvular heart disease. All procedures agree with the Declaration of Helsinki (1975 and reviewed in 1983). The study protocol was approved by the Institution’s Ethics Committee. Written informed consent was obtained prior to evaluation. 

### 2.2. Clinical Interview and Anthropometric Measurements

Before CV evaluation, a brief clinical interview together with the anthropometric and blood test results evaluation enabled assessment of exposure to CRFs. Subjects’ body weight and height were measured and body mass index (BMI) was obtained (body weight-to-square height ratio). Obesity was defined as BMI > 30 Kg/m^2^. History of dyslipidemia and diabetes were considered present if they had been previously diagnosed by referring physicians. Dyslipidemia was defined as total cholesterol (TC) >190 mg/dL, low-density lipoprotein cholesterol (LDL) >115 mg/dL, high-density lipoprotein cholesterol (HDL) for men <40 mg/dL and for women <46 mg/dL and/or triglycerides (TG) >150 mg/dL [20]. In turn, diabetes diagnosis was based on plasma glucose levels, according to American Diabetes Association criteria [21]. Hypertension (HT) was considered present if it had been previously diagnosed in agreement with reference guidelines [22]. Regular smokers (defined as usually smoking at least one cigarette/week) were identified. Family history of premature CVD was defined as a first degree relative with history of CVD before age 55 y. for men and 65 y. for women.

### 2.3. Lipid Ratios and 10. y./CVR Scores Quantification

For each subject, the CVR was quantified by means of: (1) FRS equations, (2) British National Formulary (BNF; joint British societies)-derived equation, and (3) ASSIGN equation. For instance, risk was calculated considering CVD as endpoint and 10 y. as the time period over which the risk was calculated. The risk equations included the following variables: (1) time period (10-y.), (2) age (y.), (3) sex (male/female), (4) smoking status (smoker/non-smoker) and/or number of cigarettes smoked/day, (5) diabetes (yes/no), (6) left ventricular hypertrophy on ECG (yes/no), (7) peripheral (brachial) systolic BP (pSBP; mmHg), (8) TC (mmol/L), (9) HDL (mmol/L) and/or (10) family history of premature CVD (yes/no). MS-excel versions of the equations are available [23]. Atherogenic index (AI; TC/HDL), LDL/HDL ratio and plasma atherogenic index (PAI; LogTG/HDL) were also calculated [24].

### 2.4. Cardiovascular Evaluation

Participants were asked to avoid exercise, tobacco, alcohol, caffeine, and food-intake 4h before evaluation. Measurements were done in a temperature-controlled room (21–23 °C), with the subject in supine position, after 10–15 min of rest, to achieve steady hemodynamic conditions.

#### 2.4.1. Heart Rate, Brachial, and Aortic Blood Pressure

Heart rate (HR) and peripheral (brachial) systolic and diastolic (pDBP) blood pressure levels were recorded at 8–10 min intervals using validated oscillometric devices (HEM–4030; Omron Healthcare Inc., USA). Peripheral pulse (pPP = pSBP – pDBP) and mean (pMBP = pDBP + pPP/3) blood pressure levels were calculated.

Radial artery pressure waveforms were recorded using applanation tonometry (SphygmoCor-CvMS v.9, AtCor Medical, NSW, Australia) [25]. Waves were calibrated to pDBP and pMBP and a generalized transfer function was applied to obtain central aortic blood pressure (cBP) waveforms and to quantify central systolic, diastolic and pulse (cSBP, cDBP, cPP, respectively) pressure levels. Only accurate waveforms on visual inspection and high-quality recordings (in-device quality index >75%) were considered [16].

#### 2.4.2. Carotid and Femoral Artery Ultrasound

Left and right common (CCA), internal and external carotid arteries and common femoral (CFA) arteries were examined (B-Mode and Doppler ultrasound, 7–13 MHz, linear transducer, M-Turbo, SonoSite Inc., Bothell, WA, USA) [26]. Transverse and longitudinal arterial views were obtained to assess the presence of atherosclerotic plaques. Near and far walls were analyzed, and images were obtained from anterior, lateral, and posterior angles. An atherosclerotic plaque was defined as focal wall thickening at least 50% greater than adjacent sectors, focal thickening that protrudes into the lumen at least 0.5 mm or intima-media thickness (IMT) ≥1.5 mm [26]. Plaque thickness was quantified at the site of maximal luminal infiltration, as the distance (perpendicular to the vessel wall) between the media-adventitia interface and the luminal surface of the plaque (automated procedures and digital calipers). Atherosclerotic burden was defined taking into account the number of plaques detected and territories compromised [1]. Additionally, atherosclerotic burden was defined considering dichotomous variables: 1 vs. ˃1 atherosclerotic plaques or territories with atherosclerotic plaques (carotid or femoral vs. carotid and femoral).

#### 2.4.3. Plaques’ Composition and Geometry

**Figure 1:**
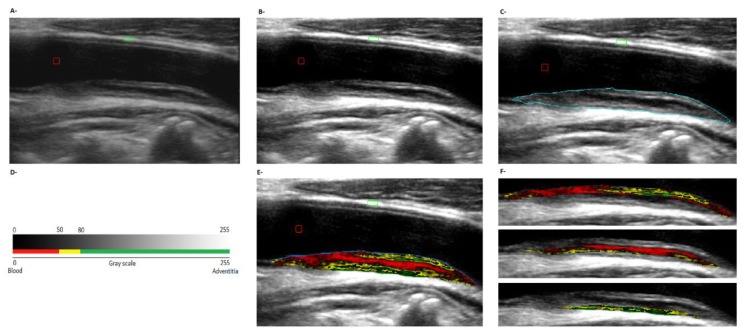
Plaque geometry and composition analysis (plaque located in the common femoral artery, posterior wall). (**A**): Raw (pre-processing) image. Red and green marks indicate, respectively the lumen (blood) and the arterial wall, references for equalization. (**B**): Equalized image. (**C**): Plaque edges determination. (**D**): Grayscale and color map. (**E**): Color map for the whole plaque. (**F**): First, second and third millimeter (sheet) analysis.

Sequences of images (videos; longitudinal axis) were stored for off-line analysis of plaques’ composition and geometric characteristics (multi-stage procedure, Hemodyn–4M, Bs.As., Argentina) [27,28]. Therefore, first a representative image of the atherosclerotic plaque was selected (Figure 1A). Second, the image was equalized (automatic linear scaling), which improved the contrast and visual appearance of the image, making it independent of operator-related characteristics and of the variability associated with differences in ultrasound gains, facilitating analysis and comparisons [13,14,28]. During equalization, the software assigned to each pixel in the image a level on a 256-level grayscale. The reference limits were previously given by the operator (0 = black, corresponding to blood; 255 = white, corresponding to the arterial wall - adventitia layer -) (Figure 1B). Third, plaque’s limits were manually defined (Figure 1C). Then, from the number of pixels within the plaque, its surface area was determined (according to calibration) [13,14,28]. Fourth, from the equalized image, the median (GSM) and mean of the plaque’s gray levels were quantified. Then, considering the GSM, plaques were ascribed to one of three groups: lipidic (GSM ≤ 50), fibrolipidic (50 < GSM ≤ 80) and fibrous (GSM > 80) [13,14] (Figure 1D). Fifth, color mapping (automatic composition analysis) assigned a color to each pixel taking into account its value in the grayscale (Figure 1E): red to pixels with values ≤50, associated with hemorrhagic and/or lipid components (and increased plaque vulnerability); yellow to pixels with grayscale values between 50 and 80, representing fibro-lipid components and green to pixels with grayscale values ˃80 related with fibrous components, as shown by histological analysis [13,14]. When a single component represented at least two thirds of the plaque, it was considered homogeneous. Otherwise, it was defined as heterogeneous [13,14]. Sixth, regional stratified grayscale analysis and color mapping were done to analyze the distribution of the different components within the plaque. To this end, the software “divided” the plaque into-one-millimeter thick sheets (from the lumen surface). Next, grayscale and color mapping analyses were done for each sheet (Figure 1E,F).

The software gave the following parameters: (a) geometrical: plaque surface (mm^2^), width (mm) and high (lumen-adventitia; mm), (b) composition: mean and median (GSM) gray levels, net and relative content of the different components (lipid, fibro-lipid, fibrous; number of pixels and %). Data about composition was obtained for the whole plaque and for each 1-mm sheet. This work considered data regarding the whole plaque and its first mm (1st mm; associated with plaque vulnerability) [29].

### 2.5. Data and Statistical Analysis

#### 2.5.1. Association between Subject Characteristics and Plaques Presence, Burden, and Characteristics

**Figure 2:**
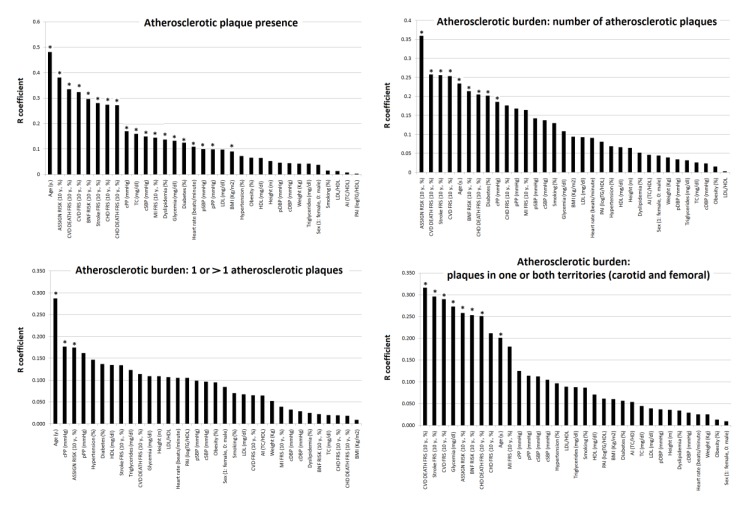
Strength of association (correlation coefficient absolute value, R) between subjects’ characteristics and plaques presence (*n =* 581) or atherosclerotic burden (*n =* 144). * *p* < 0.05.

A stepwise data analysis was done. First, variables were compared using Student’s t and chi-square test. Covariance analyses (ANCOVA) were used to compare and estimate mean differences between groups adjusting differences for age and sex (Table 1). To this end, each subject (*n* = 581) was assigned to one of two groups: with (*n* = 144) and without (*n* = 437) ultrasound-detected carotid and/or femoral atherosclerotic plaques. Second, bivariate simple and point bi-serial correlations were made in order to evaluate the associations between subjects demographic (sex, age), anthropometric (weight, height, BMI), CRFs (e.g., as continuous variables: glycemia, TC, cBP, and pBP levels; as dichotomous variables: HT, dyslipidemia, obesity, smoking), lipid ratios (TC/HDL, LDL/HDL, LogTG/HDL) and 10-y./CVR scores (e.g., 10-y. FRS for MI [10-y./FRS-MI], 10 y. FRS for CVD [10-y./FRS-CVD]) and presence of atherosclerotic plaques (*n* = 581) or atherosclerotic burden (144) (Table 2, Figure 2). We compared the strength of association between a variable (e.g., cSBP) and (a) plaque presence (yes/no) vs. (b) atherosclerotic burden (e.g., number of plaques), using William’s test for overlapping (variables in common) and dependent variables (e.g., when comparing R obtained for the association between cSBP and plaque presence vs. the association between cSBP and atherosclerotic burden) (Table 2). Third, the association between subjects’ characteristics (e.g., demographic, anthropometric, CRFs, 10-y./CVR) and those of the plaques (geometry and composition) were analyzed (*n* = 206) (Table 3). By means of William’s test for overlapping and dependent variables, we compared the association between geometric or composition variables and (1) lipid ratios (TC/HDL, LDL/HDL, LogTG/HDL), (2) 10-y./CVR (10-y./FRS-CHD, 10-y./FRS-MI, 10-y./FRS-Stroke, 10-y./FRS-CVD, 10-y./FRS-CHD DEATH, 10-y./FRS-CVD DEATH, 10-y./BNF Risk, 10-y./ASSIGN Risk) (Supplementary Tables S1–S3).

The association between exposure to CRFs (independent variables) and plaque presence (*n* = 581), atherosclerotic burden (*n* = 144) and plaques’ characteristics (*n* = 206) (dichotomous dependent variables) was evaluated using logistic regression models (LogRMs) (Supplementary Tables S4 and S5). LogRMs were constructed by introducing each CRF: (a) in isolation, (b) together with age, sex and pSBP, and (c) together with age, sex, pSBP and the remaining CRFs (Supplementary Tables S4 and S5). To evaluate dichotomously plaques characteristics, they (*n* = 206) were classified as tending to be lipidic or fibrous, depending on whether GSM or mean gray levels for the whole plaque or its 1st mm were ≤ or ˃ than the p50th. Additionally, each plaque (*n* = 206) was classified as tending to be small-medium or large, depending on whether the surface area (mm^2^) was ≤ or ˃ that the p75th of the observed values.

#### 2.5.2. Capability of Subject’s Characteristics to Detect Plaque Presence and/or Burden

Fourth, to assess the diagnostic performance or accuracy of a test (CRFs, 10-y./CVR equations and/or lipid ratios) to discriminate cases (e.g., plaque presence, elevated atherosclerotic burden) from normal subjects (e.g., plaque absence, low burden) Receiver Operating Characteristic (ROC) curve and complete sensitivity/specificity analysis were done (Table 4 and Table 5) [30]. The following parameters were quantified: area under ROC curve (AUC), Youden index, Youden index-associated criterion, sensitivity (SE), specificity (SP), positive (+LR; +LR = SE/(1-SP)) and negative likelihood ratio (-LR; -LR = (1-SE)/SP) [30,31]. Detailed data about ROC curves (criterion values and ROC curves coordinates) can be found in Supplementary Tables S6–S60 and Figures S1–55. Additionally, ROC curves were used to compare the diagnostic value of different variables (e.g., 10-y./CVR scores vs. lipid ratios) [31]. Then, CRFs, 10-y./CVR levels and lipid ratios were used as “diagnostic test” and “non-disease vs. disease” levels were considered dichotomous variables that defined plaque presence, atherosclerotic burden or plaque characteristics (Table 4 and Table 5). Data regarding ROC curves comparisons can be found in Supplementary Tables S61–S64 and Figures S56–S156.

Finally, associations between geometric and/or plaques composition characteristics (*n* = 206) were analyzed (simple and point-biserial correlations) (Table 6). Using William’s test for overlapping and dependent variables, the association of composition variables with respect to geometric parameters were analyzed comparatively (Supplementary Table S65).

According to the central limit theorem, normal distribution was considered (considering Kurtosis and Skewness coefficients distribution and number of studied subjects, with sample size ˃30) [32]. In all cases, the number of subjects and/or plaques considered in the comparisons and/or in the association analyzes (e.g., correlations) was higher than the “minimum n” calculated using an α = 0.05 and β = 0.20 (Power: 80%). Analyses were done using MedCalc Statistical Software (v.18.5, MedCalc Inc., Ostend, Belgium); Cocor Statistical Package (http://comparingcorrelations.org/) and IBM-SPSS Software (v.20, IBM-SPSS Inc., Chicago, IL, USA). A *p* < 0.05 was considered statistically significant.

## 3. Results

### 3.1. Subjects’ Characteristics

Characteristics of the entire group and of subgroups of subjects with and without plaques are shown in Table 1. Age and CRFs exposition were higher in subjects with plaques. Comparisons were done before and after statistical adjustment for covariates. pSBP, pPP, cSBP and cPP levels were higher (*p* < 0.05), whereas HR was lower (*p* = 0.009) in subjects with plaques. The differences were not significant after controlling for sex and age (Table 1). The prevalence of dyslipidemia and diabetes was higher in subjects with plaques (*p* = 0.001, *p* = 0.003). However, only the prevalence of diabetes remained different after controlling for sex and age (umbral of significance, *p* = 0.050) (Table 1). TC was higher in subjects with plaques, both before and after controlling for sex and age (*p* = 0.001 and *p* = 0.033). Glycemia was significantly higher in subjects with plaques only before controlling for age and sex. In contrast, TG levels were higher in subjects with plaques only after covariate adjustment (*p* = 0.025). All 10-y./CVR levels were higher in subjects with plaques (*p* < 0.001 and *p* < 0.01) (Table 1), but only the differences in 10-y. FRS for Stroke remained significant after adjusting for covariates.

### 3.2. Subjects’ Characteristics: Association with Plaque Presence and Atherosclerotic Burden

Age (R = 0.481), BMI (R = 0.090), pSBP (R = 0.099), cSBP (R = 0.149), pPP (R = 0.098), cPP (R = 0.170), dyslipidemia (R = 0.137), diabetes (R = 0.125), TC (R = 0.159) and glycemia (R = 0.132) were positively associated with plaque presence. All 10-y./CVR levels showed positive associations with plaque presence (R range: 0.144–0.381). HR was negatively associated with plaque presence (*p* = 0.009) (Table 2).

Age (R range: 0.201–281) was positively associated with atherosclerotic burden, disregard of the way it was defined (Table 2). The associations between other subjects’ characteristics and atherosclerotic burden varied depending on burden definition. pPP (R = 0.168), cPP (R = 0.186) and diabetes (R = 0.202) were only associated with the number of plaques. 10-y./CVR scores were not uniformly associated with atherosclerotic burden. 

With the only exception of 10-y./ASSIGN, the 10-y./CVR scores associated with atherosclerotic burden showed positive associations with the number of plaques or territories involved (i.e., one vs. both), but not with the burden defined considering the presence of 1 against more than 1 plaque (Table 2). BMI, HR, pSBP, cSBP, dyslipidemia, TC, lipid ratios, 10-y.FRS-CHD and 10-y.FRS-MI were not associated with the atherosclerotic burden (Table 2).

Considering variables associated with both plaque presence and burden it was observed that: (1) the strength of age and plaque presence association was higher than the observed between age and atherosclerotic burden (R values: 0.481 vs. 0.201, 0.234 or 0.287, *p* < 0.001), (2) regardless of the score considered, the strength of association between 10-y./CVR and atherosclerotic burden was always lower than the observed for plaque presence-risk level association, (3) diabetes (R values: 0.125 vs. 0.202, *p* = 0.003), glycemia (R values: 0.132 vs. 0.273, *p* < 0.001) and pPP (R values: 0.098 vs. 0.168, *p* = 0.007), but not cPP (0.170 vs. 0.186, *p* = 0.537) showed higher association with atherosclerosis burden than with plaque presence (depending on burden definition) (Table 2).

For plaque presence, the highest R levels were obtained forage, followed by 10-y./CVR scores (highest R levels corresponded to 10-y./ASSIGN Score) and then cPP, TC and cSBP (Figure 2). cBP showed higher levels of association than pBP (*p* < 0.05) (Figure 2). TC (but not LDL, HDL, TG or lipid ratios) achieved significant positive association with plaque presence (Table 2, Figure 2). With respect to atherosclerosis burden, and regardless of its definition, the highest Rs were found for age and 10-y./ASSIGN Score. Conversely, lipid ratios showed very low (or the lowest) R levels (Table 2, Figure 2).

Jointly analyzed results showed that age, followed by 10-y./CVR and cBP (cSBP or cPP) were the variables most strongly associated with plaque presence and atherosclerotic burden in carotid and femoral pathways. Lipid ratios showed no association with plaque presence or burden.

### 3.3. Subjects’ Characteristics: Association with Geometric Characteristics of Atherosclerotic Plaques

**Figure 3:**
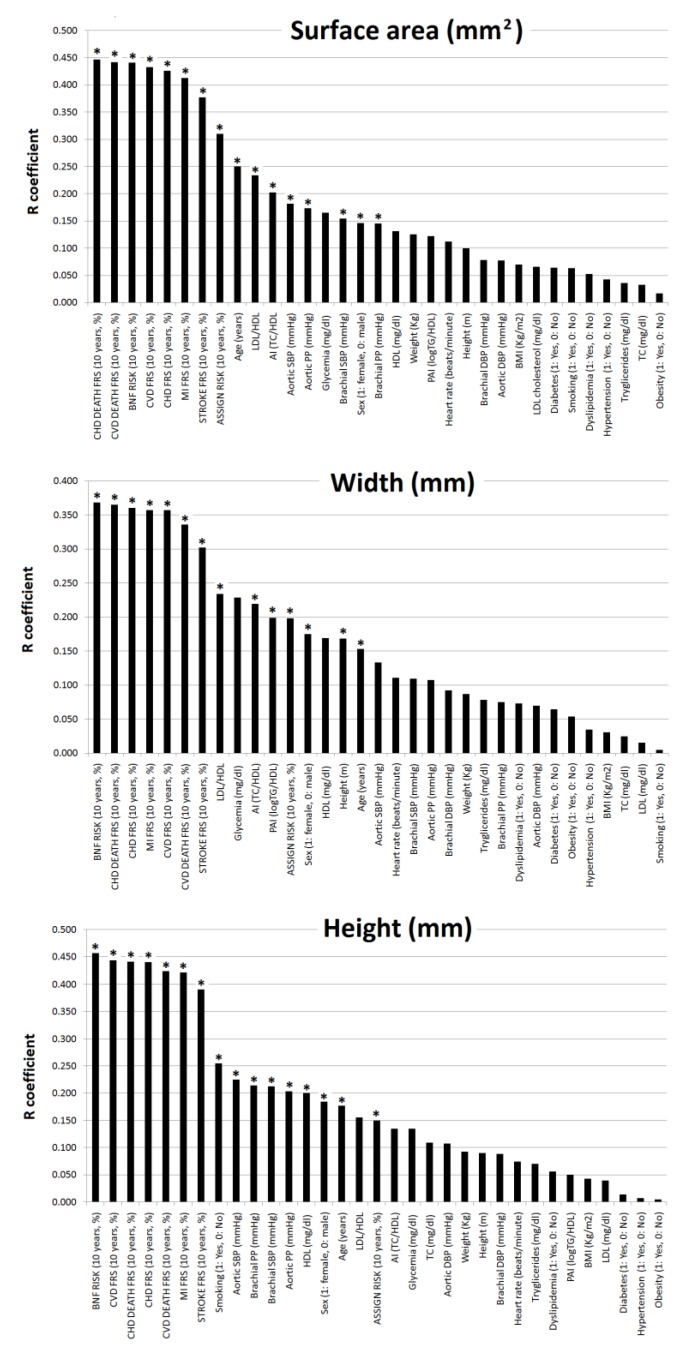
Strength of association (correlation coefficient absolute value, R) between subjects’ characteristics and plaque geometry (*n =* 206). * *p* < 0.05.

Table 3 and Figure 3 show associations between plaques’ geometric characteristics and subjects’ characteristics. Supplementary Table S1 shows the comparison of the association of plaques’ characteristics with 10-y./CVR and lipid ratios. The highest (positive) associations for the geometric characteristics (surface, width and height) corresponded to the association with 10-y./CVR. 

Plaque surface and width (but not height) showed positive associations with TC/HDL and LDL/HDL, whereas width was associated with LogTG/HDL. Sex and age showed negative and positive associations, respectively, with geometric properties. pSBP, pPP, cSBP, and cPP were positively associated with plaque surface and height, but not with its width. Compared to pBP, cBP showed higher levels of association with plaque geometry.

Geometric characteristics showed higher levels of association with 10-y./CVR than with lipid ratios (Supplementary Table S1). The strength of association varied among the 10-y./CVR scores analyzed. Geometric characteristics and lipid ratios showed progressively lower levels of association when considering LDL/HDL, TC/HDL and LogTG/HDL, respectively (Table 3, Figure 3, Supplementary Table S1).

### 3.4. Subjects’ Characteristics: Association with Plaque Composition (Fibro-Lipid Content)

**Figure 4:**
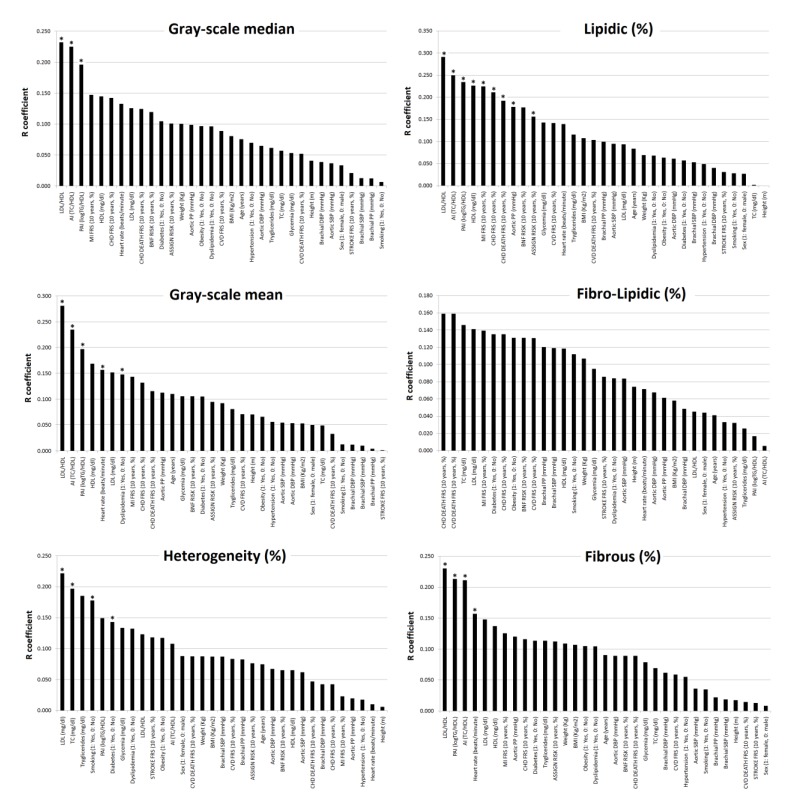
Strength of association (correlation coefficient absolute value, R) between subjects’ characteristics and plaque composition (*n =* 206). * *p* < 0.05.

**Figure 5:**
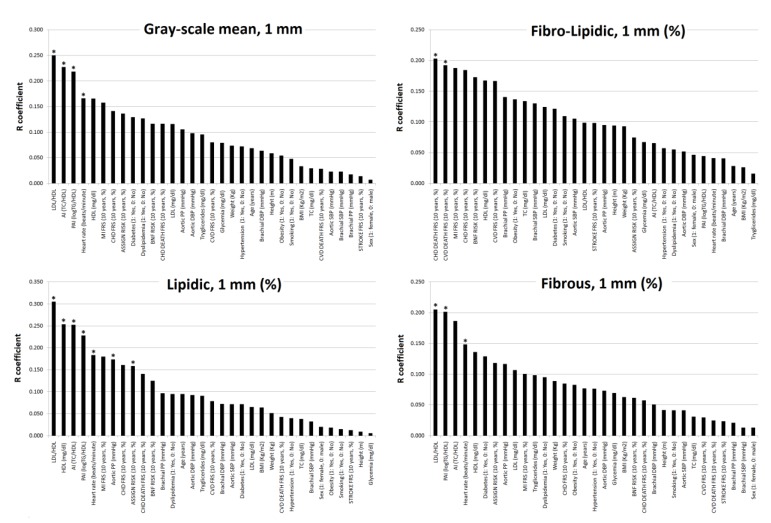
Strength of association (correlation coefficient absolute value, R) between subjects’ characteristics and composition of the plaque 1st mm (*n =* 206). * *p* < 0.05.

Table 3 and Figure 4 and Figure 5 show the associations between plaque composition and subjects’ characteristics. Supplementary Tables S2 and S3 show data related to the comparative analysis of the associations of plaque composition with lipid ratios and 10-y./CVR. TC/HDL, LDL/HDL, LogTG/HDL (mostly in that order) showed (for the whole plaque and its 1st mm): (1) negative associations with GSM, grayscale mean and fibrous content, and (2) positive associations with the lipid content (Figure 4 and Figure 5). Higher TC/HDL, LDL/HDL and/or LogTG/HDL, higher the lipid content (Table 3, Figure 4 and Figure 5).

There we no systematic or uniform associations between plaque composition and 10-y./CVR scores. There were almost no significant associations between plaque composition and CRFs (Table 3, Figure 4 and Figure 5).

LogRM analysis showed that individually, age (*p* < 0.001), pSBP (*p* = 0.018), dyslipidemia (OR = 1.9, *p* = 0.001), diabetes (OR = 5.5, *p* = 0.007) and HT (OR: 1.6, threshold of significance: *p* = 0.084) were associated with plaque presence. After adjusting by age, sex, pSBP and/or other CRFs, only the association with dyslipidemia remained statistically significant (Supplementary Table S4). Regarding atherosclerotic burden, only age showed significant association (Supplementary Table S4). None of the CRFs showed independent associations with the plaque characteristics (geometry and composition) when these were considered dichotomously (Supplementary Table S5).

### 3.5. CRFs, 10-y./CVR Scores and Lipid Ratios Capability to Detect Plaque Presence and Burden

Table 4 shows data from the analysis of ROC curves. The complete information is shown in Supplementary Tables S6–S33 and Figures S1–27.

Age (AUC: 0.820), 10-y./CVR levels (AUC range: 0.689–807) and some CRFs individually (e.g., cPP AUC: 0.628) showed statistically significant AUC for plaque presence detection. Positive detection of at least one plaque was associated with: (1) age ˃55 y. (SE/SP: 78%/74%); (2) 10-y./FRS-CVD ˃7.8% (SE/SP: 89%/59%) and (3) 10-y./ASSIGN Risk ˃9.6% (SE/SP: 71%/75%) (Table 4). In addition, for an age ˃ ~45 y. (43.19 y.) the SE/SP was 95%/38%, whereas an age ˃50 y. was associated with a SE/SP equal to 88%/60% (Supplementary Table S6). A10-y./FRS-CVD ˃~5% (5.1%) associated a SE/SP of 98%/41% to detect a plaque (Supplementary Table S18). When considering cSBP, a level ≥105 mmHg associated a SE/SP of 83%/38 (Table 4), while levels ≥110, ≥120 and ≥130 mmHg associated SE/SP equal to 63%/56%, 28%/81% and 11%/91%, respectively (Supplementary Table S11).

When analyzing the ability to distinguish the presence of one or more plaques, an age ˃58 y. was associated with a SE/SP of 85%/47%, whereas for an age ˃50 y. the SE/SP was 93.75%/20.3% (Supplementary Table S23). The SE/SP associated with a 10-y./ASSIGN ˃9.3% was 81%/40% (Table 4). None of the other scores enabled differentiation of subjects with one or more than one plaque. 

Finally, an age ˃62 y. associated an SE/SP of 69%/62 to detect compromise of both territories (carotid and femoral) (Table 4), whereas for an age ˃55 y. the SE/SP was 88.9%/30% (Supplementary Table S26). 10-y./FRS-CVD Death ˃5.2%, 10-y./FRS-Stroke ˃2.3% and 10-y./FRS-CVD ˃14.9% showed AUC ~0.7 and the best statistical ratios (Youden index) for SE/SP. Neither TC, LDL, HDL, or TG isolated levels, nor lipid ratios enabled detection of plaque presence or atherosclerotic burden (Table 4).

### 3.6. CRFs, 10-y./CVR Scores and Lipid Ratios Levels Capability to Predict Plaque Geometry and Composition

Table 5 shows data from ROC curves analysis. Supplementary Tables S34–S60 and Figures S29–S55 include complete ROC analysis. Several risk scores showed AUC ˃0.70 and cut-off levels that would enable detection of plaques with surface area ˃p75th, with high SE (˃80 or 90%) and acceptable SP (˃55%) levels (Table 5). An age ˃63 y. associated a SE/SP equal to 75.0%/59.9%, for detecting plaques with area ˃p75th; whereas SE/SP was 89.6%/24.65% for an age ˃55 y. Neither cholesterol, TG nor lipid ratios enabled detection of plaques with surfaces ˃p75th (Table 5).

Lipid ratios, particularly LDL/HDL and LogTG/HDL, enabled discrimination of whether plaque content was mostly lipidic or fibrous. LDL/HDL ˃ 2.46 associated an SE/SP of 72%/52% to identify plaques with GSM ≤ 105 and a SE/SP equal to 71%/51% to identify plaques with a grayscale media ≤110 in the 1st mm (Table 5). In turn, a LogTG/HDL ˃ 0.135 associated an SE/SP of 87%/35% to identify plaques with GSM ≤ 105, while a LogTG/HDL ˃ 0.216 showed an SE/SP equal to 68%/56% to identify plaques with a mean grayscale level ≤110 in the 1st mm. Neither TC/HDL, nor the 10-y./CVR equations were able to identify the trend of plaques’ composition (Table 5).

### 3.7. Association between Plaques’ Geometry and Composition

Geometric variables were associated (Table 6). Surface area did not show association with composition parameters. 

Plaque width was negatively associated (*p* < 0.05, or close to threshold: 0.05–0.10) with heterogeneity (R = −0.165, *p* = 0.023), GSM (R = −0.137, *p* = 0.060), mean grayscale of the plaque (R = −0.128, *p* = 0.085) and its 1st mm (R = −0.182, *p* = 0.013) and with the fibrous content of the 1st mm (R = −0.153, *p* = 0.036). 

Plaque height was positively associated with heterogeneity (R = 0.269, *p* = 0.000) and negatively associated with the fibro-lipid content of the plaque (R = −0.192, *p* = 0.008) and its 1st mm (R = −0–180, *p* = 0.013) (Table 6). 

Characteristics of the whole plaque and its 1st mm were strongly associated (e.g., grayscale mean [R = 0.964, *p* < 0.001], lipid % [R = 0.915, *p* < 0.001], fibro-lipid % [R = 0.929, *p* < 0.001] and fibrous % [R = 0.949, *p* < 0.001]) (Table 6).

## 4. Discussion

### 4.1. Main Findings

The main findings can be summarized as follows:

- First, the variables most strongly associated with the ***presence of plaques***, and which could be used to identify sub-populations that could benefit from imaging studies to individualize risk, were: age, 10-y./CVR and (although weakly) cSBP or cPP (rather than pSBP or pPP). Individually, classical CRFs and lipid ratios (TC/HDL, LDL/HDL, LogTG/HDL) showed almost no association with plaques presence. Thus, their capability to detect plaques presence would be limited.
- Second, regarding ***atherosclerotic burden***, age, and 10-y./CVR levels showed the highest levels of association. The associations with 10-y./CVR varied depending on the CVR-equation and on the definition of atherosclerotic burden considered.
- Third, ***plaques geometry*** parameters (surface, width and height) showed associations with different variables, but only 10-y./CVR followed by age and BP (mainly cBP) would allow identification of the presence of plaques with large surfaces (˃p75th).

When considering CRFs in isolation, age was the variable most strongly associated with plaque presence, atherosclerotic burden and plaque size (in that order). As expected, a higher age was positively associated with plaque presence and atherosclerotic burden. Regarding the ability to identify plaque presence, a SE/SP equal to 95%/38% was obtained when considering ≥45 y. as cut-off value, which is in line with SHAPE (Screening for Heart Attack Prevention and Education) Guidelines that proposed considering that age for the screening of carotid plaques using ultrasound [9]. It should be noted that a cut-off point a decade later (age ˃55 y.) would associate an SE/SP of 78%/74%, which should be analyzed in terms of performance and value as screening tool. When trying to identify subjects with ≥2 plaques, SE/SP values were 94%/20%, 89%/30%, 85%/47% and 69%/62% when considering as age ˃50 y., ˃55 y., ˃58 y. and ˃62 y., respectively. Similar findings and considerations apply to age cut-off values considering the associated SE/SP levels when analyzing the ability to identify plaques with surface areas ˃p75th.

Other classical CRFs (e.g., dyslipidemia, obesity) in “isolation or individually” showed almost no association with plaque presence or atherosclerosis burden, demonstrating a limited value to identify subjects with major probability of having atherosclerotic disease. Pulse and systolic (in that order) pressures were positively (although weakly) associated with plaque presence, atherosclerotic burden, and/or plaque characteristics. In almost all cases, the association levels were higher for cBP than for pBP. In this regard, previous works have shown that compared to pBP, the cBP: (1) correlates better with cardiac hypertrophy (24-h ambulatory data) [33], (2) has superior discriminatory capability to identify cardiac structural alterations (i.e., left ventricular hypertrophy and atrial dilatation), associated with (improved) reclassification in subjects with early stages of heart failure [34], (3) associates to clinical outcomes in patients with chronic kidney disease [35], (4) has enhanced association with cardiac structural features in children, adolescents and young adults [36]. The value of considering cSBP apart from pSBP has been discussed and considered over the last two decades. Our results add support to the superiority of cBP over pBP in terms of association with carotid and femoral plaque presence, atherosclerotic burden, and plaque characteristics. Looking at our findings, if cSBP were used to identify subjects with plaques, values ≥105 mmHg, ≥110 mmHg, ≥120 mmHg or ≥130 mmHg, would associate SE/SP values equal to 85%/38%, 63%/56%, 28%/81%, and 11%/91%, respectively. Then, cSBP value as a screening tool should be discussed.

Opposite to the described for CRFs when considered individually, 10-y./CVR levels were positively associated with plaque presence, plaques characteristics, and atherosclerotic burden. The levels of association varied depending on the equation considered (10-y./ASSIGN Risk Score and 10-y./FRS-CVD Death showed the strongest associations, whereas 10-y./FRS-MI showed the weakest). For plaque detection, 10-y./FRS-CVD ˃5% or ˃7.8%, showed an SE/SP of 98%/41% and 89%/59%, respectively. Regarding the ability to identify subjects with high atherosclerotic load, a 10-y./FRS-CVD ˃14.9% showed AUC ~0.7, and the best statistical ratios (Youden index), SE/SP 71%/64%, to detect involvement of both territories. In turn, 10-y./FRS-CVD ≥13.2% associated a SE/SP of 86%/51%, for the detection of plaques with a surface area ˃p75th. Consequently, 10-y./CVR levels to be considered to be cut-off values to define subjects that would benefit from further (imaging) evaluation would differ depending on the risk score selected and the aim of the screening. As an example, a value of 10-y./FRS-CVD (score widely used) ˃5% (or ˃~8%), could be used (if there is no limitation related to the number of studies to be done) as a cut-off value for screening, minimizing false negatives. A cut-off ˃13% or ˃~15% could be used to identify subjects with larger plaques and/or high atherosclerotic burden.

- Fourth, the greatest association with ***plaque composition*** was obtained for lipid ratios. Higher TC/HDL, LDL/HDL and/or LogTG/HDL levels associated higher lipid content and lower fibrous content. LDL/HDL and LogTG/HDL, but not TC/HDL, CRFs alone, nor 10-y./CVR were able to detect plaque composition.

None of the lipid ratios studied (that express the relationship between pro-atherogenic and anti-atherogenic lipids) [24] showed significant association with plaque presence or burden. Additionally, the strength of association between plaque characteristics and lipid ratios was always lower than the observed for 10-y./CVR. On the other hand, the strongest association with plaque composition was obtained for lipid ratios. Higher TC/HDL, LDL/HDL and/or LogTG/HDL associated higher lipid content, and lower fibrous content. LDL/HDL and LogTG/HDL, rather than CRFs or 10-y./CVR were able to detect plaque composition.

The higher the HDL, lower the plaque height and the lipid content of the whole plaque and its 1st mm, which could be related with the protective role ascribed to HDL in atherosclerotic disease [37]. Although HDL did not show (negative) association with plaque presence or atherosclerotic burden, higher the HDL level, lower the plaque vulnerability. It has been proposed that elevated HDL levels could contribute to plaque regression. However, there are few clinical interventional studies that causally link plasma HDL to decreased progression or to regression of atherosclerotic plaques, probably associated with the few therapeutic agents that selectively and strongly raise HDL. On the other hand, some studies have suggested that the HDL functional quality rather its levels are associated with the atherosclerotic disease [37].

As stated, lipid ratios were associated with plaque composition. Greater the ratios (i.e., greater the pro-atherogenic lipid content), lower the GSM; lower the grayscale mean (whole plaque and its 1st mm) greater the plaque lipid content and lower the fibrous content. Greater the lipid ratios, greater plaque vulnerability. Elshazly et al. demonstrated that the atherogenic index (TC/HDL) would be especially useful to identify patients who may benefit from more intensive lipid lowering to prevent disease progression and CV events, in case of discordant LDL, non-HDL-cholesterol and apoB values [38]. Ridker et al. in a prospective cohort study showed that TC/HDL would be as good as (or even better than) apo-lipid fractions to predict CV events in women after adjusting by age, smoking status, pBP, diabetes and BMI [39]. Looking at our findings, the above could be explained by the association between lipid index and plaque vulnerability. In patients treated with statins Kastelein et al. demonstrated that TC/HDL and apo-lipid B/A-I ratios were more closely associated with end-points than any of the pro-atherogenic lipid parameters considered in isolation [40]. In this work, LDL/HDL ˃2.46 associated a SE/SP of 72%/52% to identify plaques with GSM ≤105, a SE/SP equal to 73%/52% to detect plaques with mean gray levels ≤110 (and 71%/51% for a mean gray level ≤110 in the 1st mm). A LogTG/HDL ˃0.135 associated a SE/SP of 87%/35% to identify plaques with GSM or mean gray values ≤105.

- Fifth, ***the association between plaques’ geometry and composition*** was limited or non-existent.

The surface area of a plaque was not associated with its composition, but (1) wider and higher plaques were (weakly) associated with lower and higher heterogeneity levels, respectively and (2) wider plaques associated lower median and mean gray levels. The composition of a plaque and its 1st mm were strongly associated.

Our results agree with previous findings (in carotid arteries), which suggested that larger plaques (defined by their width or height), were not associated with any particular composition or vulnerability characteristics [12,41]. Studies in coronary arteries showed no association between the lipid core size and plaque surface area [42]. On the other hand, some studies showed that larger plaque volumes associated larger lipid cores [43,44]. However, those studies provided data from advanced stages in the atherosclerosis process, while our study aimed at investigating associations considering early stages of the disease. Our results are consistent with studies that proposed the value of an active preventive approach not only in case of large plaques (or those with a geometric arrangement that is likely to compromise the lumen), but in all cases, since small plaques may have significant lipid content and other characteristics associated with increased risk of plaque accidents and CV events [45,46].

Sztajzel et al. showed that low mean values on the grayscale and a high proportion of red color in the plaque superficial surface correlated with most symptoms and predicted them with higher SE and SP than whole plaque data [13,14]. In our study, the composition of the whole plaque and its 1st mm were strongly associated. Then, plaques with high lipid content were associated with a predominant lipid content in the 1st mm. Related with this, plaques would be vulnerable because of the overall lipid content and also due to the absence of a fibrous cap, associated with the lipid content prevailing in the 1st mm, characteristics that increase risk of erosions and ruptures that favor plaque accident [12,28,41]. Taking into account the above, assessing the composition of the 1st mm may be sufficient in case of limitations to determine the composition of the whole plaque.

### 4.2. Clinical Implications

Looking at our results, it could be said that due to their associations with plaque presence and burden (but not with vulnerability indexes), age and 10 y./CVR scores (with their recognized limitations) would be useful tools when assessing individual risk and defining screening in asymptomatic and non-treated subjects. In turn, lipid ratios (i.e., LDL/HDL and mainly LogTG/HDL) which were not associated with plaque presence or atherosclerotic burden, would be of value as indicators of plaque composition (vulnerability). Then, age, 10-y./CVR scores and lipid ratios should be considered complimentary at the time of assessing subject-specific CVR to define if further specific (imaging) evaluations would be of value. Carotid and femoral ultrasonographic evaluation (non-invasive, low cost and low time-consuming studies) could be considered in asymptomatic non-treated subjects aiming at: (1) population screening of atherosclerotic disease (e.g., subjects ˃45 y. and 10-y./FRS-CVD ˃5–8%); (2) identifying subjects with high atherosclerotic burden (e.g., subjects ˃50 y., 10-y./FRS-CVD ˃13–15%); (3) identifying subjects with plaques with high lipid content (e.g., subjects with LogTG/HDL ˃0.135).

### 4.3. Strengths and Limitations

Being a cross-sectional study, this work did not allow establishing causal relationships between CRFs, demographic, anthropometric, and hemodynamic characteristics with the presence or burden of subclinical atherosclerosis. Our population characteristics (e.g., age) were influenced (like in other works) by inclusion criteria. In this regard, subjects with known CVD and/or on pharmacological treatment were excluded. Since the prevalence of both conditions increases with age, old subjects included in the study may not be representative of the population of their age. Making several correlations (e.g., Table 2) increases the risk of Type I error (i.e., to erroneously conclude the presence of a significant correlation). Aiming at minimizing this, the level of statistical significance of the correlation coefficients could be adjusted (e.g., applying Bonferroni correction). However, an issue with Bonferroni correction is that it can lead to Type II errors. Analyzing pros and cons of making adjustments and the context of our research, we defined not to do them, mainly due to the following reasons. First, it would increase the risk of Type II error. As an example, in Table 2 there are 33 correlations for each variable—i.e., plaque presence—which would mean that a threshold value of p (corrected) equal to 0.0015 (0.05/33 = 0.0015) should be considered. Most of the correlations in our table showed very low *p* values (e.g., 0.003 or 0.007), which clearly have a biological theoretical explanation. Second, our results are “exploratory research/data analysis, not confirmatory”, so they were not presented with the aim of stimulating final decision making. Future work with other populations and groups should be considered before reaching definitive conclusions. Third, correlation coefficients are effect sizes, so in real terms, we do not need a p-value to interpret them. Finally, currently various techniques and biomarkers (e.g., biochemical, genetic, imaging) have been proposed to assess the characteristics of atherosclerotic plaques (e.g., to identify vulnerable plaques) [47]. Among imaging biomarkers for vulnerable plaques, echography/virtual histology, computed tomography, spectroscopy, optical coherence tomography, high-frequency intravascular ultrasound and magnetic resonance imaging were described [47]. In our work we opted for using B-Mode echography/virtual histology, since (a) we worked with peripheral plaques (non-coronary), and (b) we proposed to consider selecting a non-invasive and innocuous technique widely used in clinical practice [27,28].

## 5. Conclusions

First, the variables most strongly associated with ***plaque presence*** and which could be used to identify sub-populations that could benefit from imaging studies to individualize risk, were: age, 10-y./CVR levels and (although weakly) cSBP or cPP (rather than pSBP or pPP). Individually, classical CRFs and lipid ratios showed almost no association with plaques presence. Then, their capability to detect plaques presence would be limited. Second, age and 10-y./CVR showed the highest levels of association with ***atherosclerotic burden***, which varied depending on burden definition. Third, ***plaques geometry*** (surface, width and height) showed associations with different variables, but only 10-y./CVR followed by age and BP would allow identification of the presence of plaques with larger surfaces (˃p75th). Fourth, the greatest association with ***plaque composition*** was obtained for lipid ratios. Higher levels TC/HDL, LDL/HDL and/or LogTG/HDL associated higher lipid content and lower fibrous content. LDL/HDL and LogTG/HDL, but not the TC/HDL, CRFs alone, nor the 10-y./CVR levels were able to detect plaque composition. Fifth, in general, the ***association between plaque geometry and composition*** was scarce. There was a strong association between the composition of the whole plaque and that of its 1st mm. Carotid and femoral ultrasound evaluation (non-invasive, low cost and low time-consuming studies) could be considered in asymptomatic and non-treated subjects aiming at: (1) population screening of atherosclerotic disease (e.g., subjects ˃45y. and 10-y./FRS-CVD ˃ 5–8%); (2) identifying subjects with high atherosclerotic burden (e.g., subjects ˃50 y., 10-y./FRS-CVD ˃13–15%); (3) identifying subjects with plaques with high lipid content (e.g., subjects with LogTG/HDL ˃0.135).

## Figures and Tables

**Table 1:** Characteristics: groups comparison before and after adjustments.

	Entire Group	Atherosclerotic Plaques	Non-Atherosclerotic Plaques		
	MV ± SD	MV ± SD	MV ± SD	*p*	p*
n (% females)	581 (47.0)	144 (43.4)	437 (48.1)	0.867	-
Age (years)	51.4 ± 12.4	61.4 ± 10.0	48 ± 11.1	<0.001	-
Body weight (Kg)	76.2 ± 15.6	77.4 ± 15.9	75.9 ± 15.4	0.316	0.599
Body height (m)	1.68 ± 0.10	1.67 ± 0.10	1.68 ± 0.1	0.213	0.389
BMI (Kg/m^2^)	26.8 ± 4.5	27.5 ± 4.7	26.5 ± 4.4	0.032	0.354
Heart rate (b.p.m.)	67.04 ± 10.30	65.08 ± 9.44	67.68 ± 10.49	0.009	0.580
pSBP (mmHg)	125.78 ± 15.02	128.37 ± 14.68	124.92 ± 15.05	0.017	0.432
pDBP (mmHg)	74.75 ± 9.52	75.51 ± 9.04	74.5 ± 9.66	0.272	0.440
pPP (mmHg)	51.03 ± 10.78	52.86 ± 10.57	50.42 ± 10.8	0.018	0.694
cSBP (mmHg)	112.11 ± 13.37	115.56 ± 12.99	110.96 ± 13.31	<0.001	0.437
cDBP (mmHg)	75.74 ± 9.50	76.45 ± 9.09	75.5 ± 9.64	0.296	0.372
cPP (mmHg)	36.25 ± 8.87	38.86 ± 9.05	35.38 ± 8.65	<0.001	0.735
Hypertension (%)	14.5	18.6	12.2	**0.083**	0.265
Dyslipidemia (%)	37.1	48.3	33.2	0.001	0.056
Diabetes (%)	1.9	4.8	0.9	0.003	**0.050**
Obesity (%)	19.1	23.4	17.6	0.122	0.464
Smoking (%)	36.9	37.6	34.2	0.647	0.125
TC (mg/dL)	213.88 ± 36.96	224.71 ± 39.97	210.78 ± 35.48	0.001	0.033
HDL (mg/dL)	54.32 ± 13.04	55.63 ± 13.13	53.83 ± 13	0.255	0.256
LDL (mg/dL)	138.34 ± 33.20	143.94 ± 31.87	136.53 ± 33.41	**0.074**	0.163
AI (TC/HDL)	4.14 ± 1.11	4.16 ± 1.04	4.13 ± 1.13	0.850	0.136
LDL/HDL	2.66 ± 0.92	2.69 ± 0.79	2.66 ± 0.96	0.790	0.299
PAI (LogTG/HDL)	0.28 ± 0.26	0.28 ± 0.27	0.28 ± 0.26	0.949	0.121
TG (mg/dL)	114.00 ± 59.00	119.06 ± 76.50	112.61 ± 52.31	0.384	0.025
Glycemia (mg/dL)	90.54 ± 12.17	93.77 ± 9.70	89.74 ± 12.59	0.014	0.814
10-y./FRS-CHD (%)	7.03 ± 5.63	9.79 ± 5.44	6.17 ± 5.41	<0.001	0.573
10-y./FRS-MI (%)	3.22 ± 3.70	4.17 ± 3.39	2.92 ± 3.74	0.006	0.162
10-y./FRS-Stroke (%)	1.66 ± 2.01	2.67 ± 1.95	1.35 ± 1.93	<0.001	0.015
10-y./FRS-CVD (%)	10.42 ± 8.54	15.35 ± 7.77	8.88 ± 8.19	<0.001	0.302
10-y./FRS-CHD DEATH (%)	1.25 ± 2.01	2.23 ± 2.39	0.95 ± 1.77	<0.001	0.333
10-y./FRS-CVDDEATH (%)	2.06 ± 3.29	4.04 ± 4.02	1.45 ± 2.77	<0.001	0.466
10-y./BNF Risk Score (%)	8.69 ± 7.10	12.46 ± 6.82	7.52 ± 6.78	<0.001	0.252
10-y./ASSIGN Risk Score (%)	10.23 ± 9.59	16.70 ± 10.79	8.17 ± 8.18	<0.001	0.405

MV: mean value. SD: standard deviation. BMI: body mass index. SBP, DBP, PP: systolic, diastolic and pulse pressure (c: central [aortic]; p: peripheral [brachial]). LDL and HDL: low- and high-density lipoprotein cholesterol. TG: triglycerides. TC: total cholesterol. AI: atherogenic index. PAI: plasma atherogenic index. y.: years. FRS: Framingham Risk Score. CHD: coronary heart disease. MI: myocardial infarction. CVD: cardiovascular disease. BNF: British National Foundation Score Risk. *p* and p*: *p*-value before and after adjusted for sex = 0.47 and age = 51.42 (ANCOVA).

**Table 2:** Associations between subjects’ characteristics and plaques presence or burden: correlation comparison.

		Presence (1)	Burden (2)	Burden (3)	Burden (4)	Correlation Comparison (William’s Test)					
		(*n =* 581)	(*n =* 144)	(*n =* 144)	(*n =* 144)	1 vs. 2		1 vs. 3		1 vs. 4	
		1: yes, 0: no	Nº of AP	Nº AP, 1 or >1	Nº territories	ΔR	*p*	ΔR	*p*	ΔR	*p*
Sex (1: female, 0: male)	R	−0.037	0.045	0.085	0.009	−0.082	**0.002**	−0.122	**<0.001**	−0.047	**0.004**
	P	0.370	0.596	0.314	0.911						
Age (y.)	R	0.481	0.234	0.287	0.201	0.247	**0.000**	0.194	**<0.001**	0.280	**<0.001**
	P	**<0.001**	**0.005**	**<0.001**	**0.015**						
Weight (Kg)	R	0.042	0.040	−0.052	0.025	0.003	0.940	0.095	**<0.001**	0.017	0.287
	P	0.314	0.639	0.539	0.768						
Height (m)	R	−0.053	−0.065	−0.109	−0.036	0.012	0.649	0.056	**<0.001**	−0.017	0.287
	P	0.208	0.447	0.198	0.674						
BMI (Kg/m^2^)	R	0.090	0.094	0.009	0.060	−0.004	0.879	0.081	**<0.001**	0.030	**0.060**
	P	**0.031**	0.268	0.912	0.476						
Heart rate (b.p.m.)	R	−0.109	−0.091	−0.106	−0.025	−0.018	0.493	−0.003	0.836	−0.084	**<0.001**
	P	**0.009**	0.282	0.210	0.765						
pSBP (mmHg)	R	0.099	0.142	0.099	0.105	−0.043	0.100	0.000	1.000	−0.006	0.706
	P	**0.017**	**0.089**	0.238	0.211						
pDBP (mmHg)	R	0.046	0.034	−0.029	0.037	0.011	0.649	0.074	**<0.001**	0.009	0.573
	P	0.272	0.684	0.732	0.661						
pPP (mmHg)	R	0.098	0.168	0.162	0.114	−0.070	**0.007**	−0.064	**<0.001**	−0.016	**0.314**
	P	**0.018**	**0.044**	**0.052**	0.173						
cSBP (mmHg)	R	0.149	0.138	0.097	0.112	0.011	0.673	0.052	**<0.001**	0.037	**0.019**
	P	**<0.001**	**0.100**	0.249	0.180						
cDBP (mmHg)	R	0.044	0.024	−0.033	0.029	0.020	0.448	0.076	**<0.001**	0.014	0.348
	P	0.296	0.776	0.698	0.728						
cPP (mmHg)	R	0.170	0.186	0.177	0.125	−0.016	0.537	−0.007	0.627	0.045	**0.004**
	P	**<0.001**	**0.025**	**0.034**	0.135						
Hypertension	R	0.072	0.069	0.147	0.096	0.004	0.909	−0.074	**<0.001**	−0.024	0.132
	P	**0.083**	0.414	**0.080**	0.252						
Dyslipidemia	R	0.137	0.052	−0.025	0.034	0.085	**0.001**	0.162	**<0.001**	0.103	**<0.001**
	P	**0.001**	0.533	0.767	0.688						
Diabetes	R	0.125	0.202	0.137	0.057	−0.077	**0.003**	−0.012	0.408	0.068	**<0.001**
	P	**0.003**	**0.015**	0.101	0.500						
Obesity	R	0.066	0.015	−0.095	0.013	0.050	0.053	0.161	**<0.001**	0.053	**0.001**
	P	0.113	0.854	0.257	0.875						
Smoking	R	0.015	0.130	0.070	0.087	−0.114	**<0.001**	−0.055	**0.001**	−0.071	**<0.001**
	P	0.715	0.121	0.402	0.301						
TC (mg/dL)	R	0.159	−0.027	0.020	0.045	0.186	**<0.001**	0.139	**<0.001**	0.114	**<0.001**
	P	**0.001**	0.800	0.847	0.671						
		Presence (1)	Burden (2)	Burden (3)	Burden (4)	Correlation comparison (William’s Test)					
		(*n =* 581)	(*n =* 144)	(*n =* 144)	(*n =* 144)	1 vs. 2		1 vs. 3		1 vs. 4	
		1: yes, 0: no	Nº of AP	Nº AP, 1 or >1	Nº territories	ΔR	*p*	ΔR	*P*	ΔR	*P*
HDL (mg/dL)	R	0.065	−0.066	0.135	0.071	0.131	**<0.001**	−0.070	**<0.001**	−0.006	0.707
	P	0.219	0.535	0.206	0.507						
LDL (mg/dL)	R	0.098	−0.093	−0.067	−0.039	0.190	**<0.001**	0.165	**<0.001**	0.137	**<0.001**
	P	0.067	0.405	0.545	0.726						
AI (TC/HDL)	R	0.007	0.046	−0.065	−0.054	−0.039	0.139	0.072	**<0.001**	0.061	**<0.001**
	P	0.892	0.667	0.546	0.616						
LDL/HDL	R	0.013	0.003	−0.107	−0.089	0.010	0.851	0.120	**<0.001**	0.102	**<0.001**
	P	0.809	0.977	0.335	0.425						
PAI (LogTG/HDL)	R	−0.003	0.081	−0.105	−0.062	−0.084	**0.014**	0.102	**<0.001**	0.059	**<0.001**
	P	0.949	0.468	0.343	0.580						
TG (mg/dL)	R	0.042	−0.032	−0.124	−0.088	0.074	**0.005**	0.166	**<0.001**	0.129	**<0.001**
	P	0.437	0.772	0.266	0.431						
Glycemia (mg/dL)	R	0.132	0.109	0.109	0.273	0.023	0.379	0.023	0.113	−0.141	**<0.001**
	P	**0.014**	0.378	0.376	**0.024**						
10-y./FRS-CHD (%)	R	0.274	0.177	−0.020	0.211	0.097	**<0.001**	0.294	**<0.001**	0.063	**<0.001**
	P	**<0.001**	0.106	0.856	**0.052**						
10-y./FRS-MI (%)	R	0.144	0.164	−0.039	0.181	−0.020	0.442	0.183	**<0.001**	−0.037	**0.019**
	P	**0.006**	0.133	0.720	**0.098**						
10-y./FRS-Stroke (%)	R	0.280	0.256	0.135	0.296	0.024	0.192	0.145	**<0.001**	−0.016	0.295
	P	**<0.001**	**0.018**	0.220	**0.006**						
10-y./FRS-CVD (%)	R	0.323	0.254	0.065	0.290	0.069	**0.006**	0.258	**<0.001**	0.033	**0.029**
	P	**<0.001**	**0.019**	0.552	**0.007**						
10-y./FRS-CHD DEATH (%)	R	0.272	0.206	0.019	0.251	0.066	**0.010**	0.253	**<0.001**	0.021	0.173
	P	**<0.001**	**0.059**	0.865	**0.021**						
10-y./FRS-CVD DEATH (%)	R	0.335	0.258	0.114	0.316	0.077	**0.021**	0.221	**<0.001**	0.019	0.208
	P	**<0.001**	**0.017**	0.299	**0.003**						
10-y./BNF Risk Score (%)	R	0.296	0.214	0.023	0.253	0.082	**0.001**	0.273	**<0.001**	0.043	**0.005**
	P	**<0.001**	**0.049**	0.838	**0.019**						
10-y./ASSIGN Risk Score (%)	R	0.381	0.360	0.175	0.258	0.021	0.387	0.206	**<0.001**	0.123	**<0.001**
	p	**<0.001**	**<0.001**	**0.041**	**0.002**						

BMI: body mass index. SBP, DBP, PP: systolic, diastolic, and pulse pressure (c: central; p: peripheral [brachial]). LDL and HDL: low- and high-density lipoprotein cholesterol. TC: total cholesterol. TG: triglycerides. AI: atherogenic index. PAI: plasma atherogenic index. y.: years. FRS: Framingham Risk Score. CHD: coronary heart disease. MI: myocardial infarction. CVD: cardiovascular disease. BNF: British National Foundation. Cardiovascular risk factors: 1: yes (presence), 0: no. AP: atherosclerotic plaques. Nº of AP: number of AP. Nº territories: affected territories (one or both). ΔR: difference between R coefficients. *p*: *p*-value.

**Table 3:** Association of plaques’ geometry and composition with subjects’ demographic, anthropometric, hemodynamic and cardiovascular risk characteristics (*n =* 206).

	Surface Area (mm^2^)		Width (mm)		Height (mm)		Grayscale Median		Grayscale Mean		Heterogeneity %			
	R	*p*	R	*P*	R	*p*	R	*p*	R	*p*	R		*p*	
Sex (1: female, 0: male)	−0.146	**0.044**	−0.175	**0.016**	−0.185	**0.009**	0.034	0.643	0.050	0.500	−0.088		0.227	
Age (y.)	0.250	**0.001**	0.153	**0.035**	0.177	**0.012**	0.076	0.296	0.110	0.136	0.075		0.304	
Weight (Kg)	0.125	**0.090**	0.087	0.241	0.093	0.195	−0.100	0.17	−0.092	0.218	−0.087		0.236	
Height (m)	0.099	0.178	0.168	**0.023**	0.090	0.209	−0.041	0.577	−0.070	0.347	−0.006		0.937	
BMI (Kg/m^2^)	0.070	0.344	−0.030	0.681	0.043	0.552	−0.081	0.271	−0.053	0.481	−0.087		0.237	
Heart rate (b.p.m.)	−0.112	0.127	−0.111	0.130	−0.075	0.295	0.133	**0.067**	0.157	**0.034**	0.010		0.890	
pSBP (mmHg)	0.154	**0.033**	0.109	0.134	0.213	**0.002**	0.013	0.86	0.010	0.890	0.087		0.232	
pDBP (mmHg)	0.078	0.286	0.092	0.205	0.089	0.211	0.039	0.589	0.012	0.867	0.042		0.560	
pPP (mmHg)	0.145	**0.046**	0.075	0.306	0.214	**0.002**	−0.012	0.864	0.004	0.953	0.083		0.256	
cSBP (mmHg)	0.182	**0.012**	0.133	0.067	0.225	**0.001**	−0.037	0.612	−0.055	0.460	0.062		0.394	
cDBP (mmHg)	0.077	0.292	0.070	0.338	0.108	0.129	0.065	0.368	0.054	0.468	0.067		0.356	
cPP (mmHg)	0.173	**0.017**	0.107	0.142	0.204	**0.004**	−0.099	0.171	−0.113	0.126	0.020		0.787	
Hypertension	0.043	0.559	0.035	0.635	0.008	0.915	−0.070	0.334	−0.056	0.448	−0.018		0.809	
Dyslipidemia	0.052	0.476	0.073	0.318	0.056	0.429	−0.096	0.182	−0.148	**0.045**	−0.132		**0.068**	
Diabetes	0.063	0.384	0.064	0.379	−0.014	0.846	−0.105	0.148	−0.105	0.154	−0.143		**0.048**	
Obesity	−0.017	0.819	−0.054	0.468	0.005	0.947	−0.097	0.186	−0.066	0.379	−0.117		0.111	
Smoking	−0.063	0.388	−0.005	0.947	−0.255	<0.001	−0.007	0.926	−0.013	0.859	−0.178		**0.014**	
TC (mg/dL)	0.033	0.734	−0.025	0.798	−0.110	0.240	−0.057	0.548	−0.049	0.607	−0.197		**0.038**	
HDL (mg/dL)	−0.131	0.180	−0.169	0.082	−0.200	0.034	0.145	0.131	0.169	0.081	−0.065		0.503	
LDL (mg/dL)	0.065	0.524	0.015	0.884	−0.040	0.688	−0.126	0.215	−0.152	0.137	−0.222		**0.029**	
AI (TC/HDL)	0.202	**0.038**	0.219	**0.024**	0.135	0.156	−0.225	**0.019**	−0.235	**0.015**	−0.108		0.270	
LDL/HDL	0.234	**0.021**	0.234	**0.021**	0.156	0.117	−0.232	**0.021**	−0.281	**0.005**	−0.123		0.229	
PAI (LogTG/HDL)	0.122	0.232	0.199	**0.049**	0.051	0.610	−0.196	**0.049**	−0.197	**0.050**	−0.149		0.141	
Triglycerides (mg/dL)	0.036	0.727	0.078	0.444	−0.070	0.478	−0.062	0.539	−0.081	0.422	−0.185		**0.067**	
Glycemia (mg/dL)	0.165	0.243	0.228	0.103	0.135	0.326	−0.053	0.705	−0.106	0.454	−0.133		0.345	
10-y./FRS-CHD (%)	0.426	**<0.001**	0.36	**<0.001**	0.44	**<0.001**	−0.142	0.14	−0.132	0.175	0.042		0.666	
10-y./FRS-MI (%)	0.413	**<0.001**	0.357	**<0.001**	0.421	**<0.001**	−0.147	0.127	−0.144	0.140	0.023		0.816	
10-y./FRS-Stroke(%)	0.377	**<0.001**	0.302	**0.002**	0.391	**<0.001**	−0.021	0.827	0.001	0.989	0.118		0.225	
10-y./FRS-CVD (%)	0.433	**<0.001**	0.357	**<0.001**	0.444	**<0.001**	−0.089	0.358	−0.071	0.466	0.083		0.393	
10-y./FRS-CHD DEATH (%)	0.447	**<0.001**	0.365	**<0.001**	0.441	**<0.001**	−0.125	0.197	−0.116	0.236	0.047		0.631	
10-y./FRS-CVD DEATH (%)	0.442	**<0.001**	0.336	**<0.001**	0.424	**<0.001**	−0.052	0.591	−0.033	0.733	0.087		0.370	
10-y./BNF Risk Score (%)	0.441	**<0.001**	0.368	**<0.001**	0.457	**<0.001**	−0.120	0.215	−0.106	0.278	0.065		0.505	
10-y./ASSIGN Risk Score (%)	0.31	**<0.001**	0.198	**0.007**	0.150	**0.038**	−0.101	0.166	−0.095	0.205	−0.076		0.299	
	Lipidic (%)		Fibrolipidic (%)		Fibrous (%)		Grayscale mean, 1 mm		Lipidic, 1 mm (%)		Fibrolipidic, 1 mm (%)		Fibrous, 1 mm (%)	
	R	*p*	R	*p*	R	*p*	R	*p*	R	*p*	R	*P*	R	*p*
Sex	−0.027	0.715	0.044	0.547	0.008	0.911	0.007	0.925	−0.020	0.789	0.046	0.528	−0.013	0.862
Age (y.)	−0.083	0.254	−0.041	0.575	0.090	0.216	0.068	0.353	−0.094	0.199	−0.028	0.703	0.076	0.297
Weight (Kg)	0.069	0.350	0.107	0.148	−0.109	0.141	−0.074	0.323	0.051	0.494	0.093	0.211	−0.088	0.234
Height (m)	−0.035	0.641	0.074	0.317	−0.018	0.812	−0.058	0.433	−0.009	0.909	0.094	0.207	−0.041	0.578
BMI (Kg/m^2^)	0.107	0.148	0.058	0.434	−0.107	0.147	−0.033	0.654	0.064	0.392	0.026	0.725	−0.062	0.402
HR (b.p.m.)	−0.139	0.058	−0.071	0.332	0.157	**0.031**	0.166	**0.024**	−0.183	**0.013**	−0.041	0.578	0.148	**0.044**
pSBP (mmHg)	0.053	0.472	−0.119	0.102	0.019	0.799	0.023	0.759	0.032	0.663	−0.130	**0.076**	0.013	0.860
pDBP (mmHg)	−0.040	0.584	−0.048	0.508	0.062	0.396	0.064	0.387	−0.072	0.327	−0.040	0.582	0.050	0.492
pPP (mmHg)	0.099	0.174	−0.120	**0.099**	−0.022	0.766	−0.017	0.813	0.096	0.19	−0.140	**0.055**	−0.021	0.776
cSBP (mmHg)	0.095	0.195	−0.084	0.252	−0.036	0.623	−0.023	0.756	0.071	0.333	−0.105	0.151	−0.041	0.576
cDBP (mmHg)	−0.061	0.404	−0.067	0.355	0.089	0.221	0.098	0.185	−0.092	0.210	−0.052	0.481	0.073	0.319
cPP (mmHg)	0.178	**0.014**	−0.061	0.403	−0.120	**0.100**	−0.106	0.152	0.173	**0.018**	−0.095	0.196	−0.116	0.112
Hypertension	0.049	0.505	0.033	0.653	−0.055	0.449	−0.072	0.330	0.039	0.595	0.057	0.438	−0.076	0.298
Dyslipidemia	0.068	0.353	0.084	0.250	−0.104	0.151	−0.127	**0.085**	0.095	0.198	0.055	0.452	−0.095	0.196
Diabetes	0.057	0.437	0.135	**0.063**	−0.114	0.119	−0.129	**0.079**	0.071	0.333	0.121	**0.097**	−0.129	0.079
Obesity	0.063	0.395	0.131	**0.076**	−0.105	0.156	−0.054	0.468	0.018	0.810	0.137	**0.065**	−0.082	0.268
Smoking	−0.028	0.707	0.112	0.125	−0.035	0.635	−0.048	0.517	0.014	0.845	0.109	0.136	−0.041	0.575
TC (mg/dL)	0.002	0.987	0.146	0.129	−0.069	0.474	−0.029	0.764	−0.038	0.698	0.134	0.165	−0.031	0.750
HDL (mg/dL)	−0.226	**0.019**	0.118	0.225	0.137	0.159	0.165	**0.092**	−0.253	**0.009**	0.167	**0.087**	0.136	0.165
LDL (mg/dL)	0.093	0.365	0.141	0.168	−0.148	0.148	−0.116	0.265	0.065	0.530	0.124	0.228	−0.106	0.302
AI (TC/HDL)	0.249	**0.010**	−0.005	0.955	−0.211	**0.030**	−0.227	**0.021**	0.252	**0.010**	−0.065	0.508	−0.186	**0.057**
LDL/HDL	0.291	**0.004**	−0.045	0.661	−0.230	**0.024**	−0.250	**0.014**	0.305	**0.003**	−0.098	0.340	−0.205	**0.045**
PAI (LogTG/HDL)	0.234	**0.020**	0.017	0.870	−0.213	**0.036**	−0.218	**0.033**	0.228	**0.024**	−0.044	0.667	−0.201	**0.048**
TG (mg/dL)	0.115	0.258	0.026	0.803	−0.113	0.266	−0.095	0.356	0.090	0.378	−0.016	0.879	−0.098	0.338
Glycemia (mg/dL)	0.142	0.314	−0.095	0.504	−0.079	0.580	−0.079	0.581	0.005	0.971	−0.067	0.640	−0.069	0.629
10-y./FRS-CHD (%)	0.211	**0.030**	−0.135	0.168	−0.116	0.238	−0.141	0.153	0.160	0.102	−0.184	**0.060**	−0.085	0.391
10-y./FRS-MI (%)	0.224	**0.021**	−0.139	0.155	−0.126	0.200	−0.157	0.110	0.179	**0.067**	−0.188	**0.055**	−0.100	0.309
10-y./FRS-Stroke ( %)	0.031	0.753	−0.085	0.384	0.0130	0.896	−0.014	0.889	−0.012	0.901	−0.098	0.320	0.023	0.816
10-y./FRS-CVD (%)	0.142	0.148	−0.130	0.183	−0.059	0.548	−0.080	0.420	0.078	0.428	−0.166	**0.090**	−0.030	0.765
10-y./FRS-CHD DEATH (%)	0.192	**0.049**	−0.159	0.104	−0.089	0.364	−0.116	0.240	0.14	0.154	−0.203	**0.038**	−0.057	0.561
10-y./FRS-CVD DEATH (%)	0.103	0.292	−0.159	0.104	−0.015	0.883	−0.028	0.776	0.042	0.667	−0.192	**0.050**	0.024	0.805
10-y./BNF Risk Score (%)	0.177	**0.070**	−0.131	0.182	−0.089	0.364	−0.116	0.240	0.125	0.205	−0.173	**0.078**	−0.061	0.534
10-y./ASSIGN Risk Score (%)	0.156	**0.034**	−0.032	0.664	−0.112	0.128	−0.136	**0.067**	0.158	**0.033**	−0.075	0.315	−0.118	0.11

BMI: body mass index. SBP, DBP, PP: systolic, diastolic and pulse pressure (c: central; p: peripheral [brachial]). HR: heart rate. LDL and HDL: low- and high-density lipoprotein. TC: total cholesterol. TG: triglycerides. Sex: 1: female, 0: male. AI: atherogenic index. PAI: plasma atherogenic index. y.: years. FRS: Framingham Risk Score. CHD: coronary heart disease. MI: myocardial infarction. CVD: cardiovascular disease. BNF: British National Foundation. Cardiovascular risk factors: 1: yes (presence), 0: no. *p*: *p*-value.

**Table 4:** Atherosclerotic plaques presence or burden: ROC curves-derived parameters.

	AUC	AUC 95% CI	AUC p	Youden Index (J)	Associated Criterion	SE	SP	+LR	-LR
**Presence of atherosclerotic plaques (1: yes, 0: no) (*n =* 581)**									
Age (y.)	0.82	0.78–0.85	**<0.001**	0.519	>54.9	77.78	74.14	3.01	0.30
10-y./FRS-CVD DEATH (%)	0.81	0.76–0.84	**<0.001**	0.474	>0.91	81.18	64.47	2.28	0.29
10-y./ASSIGN Risk Score (%)	0.80	0.76–0.83	**<0.001**	0.4656	>9.60	71.32	75.23	2.88	0.38
10-y./FRS-Stroke (%)	0.79	0.74–0.83	**<0.001**	0.4858	>1.24	81.18	67.40	2.49	0.28
10-y./FRS-CHD DEATH (%)	0.79	0.73–0.82	**<0.001**	0.489	>0.57	81.18	67.77	2.52	0.28
10-y./FRS-CVD (%)	0.78	0.73–0.82	**<0.001**	0.4802	>7.81	89.41	58.61	2.16	0.18
10-y./BNF Risk Score (%)	0.76	0.71–0.80	**<0.001**	0.4582	>6.45	89.41	56.41	2.05	0.19
10-y./FRS-CHD (%)	0.74	0.69–0.78	**<0.001**	0.4362	>5.15	89.41	54.21	1.95	0.20
10-y./FRS-MI (%)	0.69	0.63–0.73	**<0.001**	0.3369	>1.86	77.65	56.04	1.77	0.40
cPP (mmHg)	0.63	0.58–0.66	**<0.001**	0.211	>35	62.50	58.60	1.51	0.64
TC (mg/dL)	0.62	0.57–0.66	**<0.001**	0.236	>216	61.29	62.35	1.63	0.62
cSBP (mmHg)	0.62	0.57–0.65	**<0.001**	0.208	>105	82.64	38.14	1.34	0.46
Glycemia (mg/dL)	0.61	0.55–0.65	**0.005**	0.196	>87	79.41	40.22	1.33	0.51
pPP (mmHg)	0.58	0.53–0.62	**0.003**	0.171	>45	78.47	38.67	1.28	0.56
pSBP (mmHg)	0.58	0.53–0.61	**0.005**	0.145	>128	47.22	67.28	1.44	0.78
HR (b.p.m.)	0.57	0.52–0.60	**0.015**	0.109	<63	47.55	63.39	1.30	0.83
BMI (Kg/m^2^)	0.56	0.51–0.59	**0.040**	0.129	>25.80	63.83	49.06	1.25	0.74
**Atherosclerotic burden (Nº of plaques, 1 or >1) (*n =* 144)**									
Age (y.).	0.66	0.58–0.74	**<0.001**	0.319	>58.18	85.00	46.88	1.60	0.32
10-y./ASSIGN Risk Score (%)	0.61	0.52–0.69	**0.026**	0.205	>9.30	80.82	39.68	1.34	0.48
cPP (mmHg)	0.58	0.49–0.66	**0.080**	0.159	>34	73.75	42.19	1.28	0.62
**Atherosclerotic burden (Nº of arterial territories, 1 or >1) (*n =* 144)**									
10-y./FRS-CVD DEATH (%)	0.70	0.58–0.79	**0.004**	0.336	>5.23	52.38	81.25	2.79	0.59
10-y./FRS-Stroke (%)	0.70	0.58–0.79	**0.004**	0.354	>2.33	66.67	68.75	2.13	0.48
10-y./FRS-CVD (%)	0.69	0.58–0.78	**0.003**	0.355	>14.88	71.43	64.06	1.99	0.45
10-y./BNF Risk Score (%)	0.67	0.555–0.765	**0.011**	0.250	>7.148	100.00	25.00	1.33	0.00
10-y./FRS-CHD DEATH (%)	0.66	0.547–0.757	**0.023**	0.257	>2.362	47.62	78.12	2.18	0.67
Glycemia (mg/dL)	0.65	0.528–0.765	**0.065**	0.331	>95	61.11	72.00	2.18	0.54
10-y./ASSIGN Risk Score (%)	0.64	0.55–0.71	**0.009**	0.243	>8.31	92.68	31.58	1.35	0.23
Age (y.)	0.63	0.54–0.71	**0.006**	0.305	>61.94	68.89	61.62	1.79	0.50

AUC: Area under the ROC curve. AUC 95% CI: AUC 95% confidence interval. AUC p: AUC significance level P (Area = 0.5). SE: sensitivity. SP: Specificity. +LR: Positive likelihood ratio. -LR: Negative likelihood ratio. BMI: body mass index. SBP, PP: systolic and pulse pressure (c: central; p: peripheral [brachial]). HR: heart rate. TC: total cholesterol. y.: years. FRS: Framingham Risk Score. CHD: coronary heart disease. MI: myocardial infarction. CVD: cardiovascular disease. BNF: British National Foundation.

**Table 5:** Plaques geometry and composition detection: ROC curves-derived parameters.

	AUC	AUC 95% CI	AUC p	Youden Index(J)	Associated Criterion	SE	SP	+LR	-LR
**Surface area (mm^2^) (<p75th or ≥p75th) (*n =* 206)**									
10-y./FRS-Stroke (%)	0.73	0.63–0.81	**<0.001**	0.386	>3.302	64.29	74.36	2.51	0.48
10-y./FRS-CVD DEATH (%)	0.72	0.62–0.80	**<0.001**	0.321	>5.960	50.00	82.05	2.79	0.61
10-y./FRS-CVD (%)	0.71	0.61–0.79	**<0.001**	0.370	>13.231	85.71	51.28	1.76	0.28
10-y./FRS-CHD DEATH (%)	0.71	0.61–0.79	**<0.001**	0.365	>0.988	92.86	43.59	1.65	0.16
10-y./BNF Risk Score (%)	0.71	0.61–0.79	**<0.001**	0.370	>10.310	85.71	51.28	1.76	0.28
Age (y.)	0.69	0.62–0.75	**<0.001**	0.349	>63.705	75.00	59.86	1.87	0.42
10-y./ASSIGN Risk Score (%)	0.68	0.60–0.74	**<0.001**	0.292	>9.750	93.48	35.71	1.45	0.18
10-y./FRS-CHD (%)	0.67	0.57–0.75	**0.006**	0.283	>7.141	82.14	46.15	1.53	0.39
cPP (mmHg)	0.66	0.58–0.72	**<0.001**	0.328	>39	72.92	59.86	1.82	0.45
10-y./FRS-MI (%)	0.65	0.54–0.73	**0.021**	0.242	>8.293	35.71	88.46	3.10	0.73
pPP (mmHg)	0.63	0.56–0.70	**0.003**	0.213	>62	39.58	81.69	2.16	0.74
cSBP (mmHg)	0.62	0.54–0.68	**0.016**	0.211	>122	52.08	69.01	1.68	0.69
pSBP (mmHg)	0.59	0.51–0.660	**0.060**	0.172	>144	31.25	85.92	2.22	0.80
LDL/HDL	0.56	0.45–0.65	**0.442**	0.194	>2.970	46.15	73.24	1.72	0.74
AI (TC/HDL)	0.55	0.45–0.65	**0.413**	0.200	>3.936	75.86	44.16	1.36	0.55
**Plaque composition (0: fibrous, 1: lipidic + fibrolipidic) (*n =* 206)**									
PAI (LogTG/HDL)	0.58	0.50–0.65	**0.067**	0.207	>0.301	53.03	67.68	1.64	0.69
LDL/HDL	0.55	0.47–0.62	0.275	0.146	>2.590	54.55	60.00	1.36	0.76
AI (TC/HDL)	0.53	0.44–0.60	0.584	0.102	>4.300	39.13	71.03	1.35	0.86
**Plaque composition (GSM ≤105 or >105) (*n =* 206)**									
LDL/HDL	0.61	0.51–0.70	**0.044**	0.244	>2.461	72.41	52.00	1.51	0.53
PAI (LogTG/HDL)	0.61	0.50–0.71	**0.052**	0.220	>0.135	87.27	34.78	1.34	0.37
AI (TC/HDL)	0.58	0.47- 0.67	0.177	0.186	>3.432	90.00	28.57	1.26	0.35
**Plaque composition (Media grayscale ≤110 or >110) (*n =* 206)**									
LDL/HDL	0.61	0.51–071	**0.040**	0.252	>2.462	73.21	52.00	1.53	0.52
PAI (LogTG/HDL)	0.60	0.50–0.69	**0.085**	0.218	>0.135	87.04	34.78	1.33	0.37
AI (TC/HDL)	0.56	0.48- 0.67	0.182	0.200	>3.433	91.38	28.57	1.28	0.3
**Plaque composition (Media grayscale 1mm ≤110 or >110) (** ***n =*** **206)**									
PAI (LogTG/HDL)	0.64	0.53–0.73	**0.016**	0.237	>0.216	67.92	55.81	1.54	0.57
LDL/HDL	0.63	0.53–0.72	**0.018**	0.224	>2.461	71.43	51.06	1.46	0.56
AI (TC/HDL)	0.58	0.48–0.67	**0.142**	0.182	>4.3	46.55	71.74	1.65	0.75

AUC: Area under the ROC curve. AUC 95%CI: AUC 95% confidence interval. AUC p: AUC significance level P (Area = 0.5). SE: Sensitivity. SP: Specificity. +LR: Positive likelihood ratio. -LR: Negative likelihood ratio. SBP, PP: systolic and pulse pressure (c: central; p: peripheral [brachial]). LDL and HDL: low- and high-density lipoprotein. TC: total cholesterol. TG: triglycerides. y.: years. FRS: Framingham Risk Score. CHD: coronary heart disease. MI: myocardial infarction. CVD: cardiovascular disease. BNF: British National Foundation. AI: atherogenic index. PAI: plasma atherogenic index. GSM: grayscale median.

**Table 6:** Bivariate association between plaques geometry and composition **(*n =* 206)**.

	Surface Area (mm^2^)		Width (mm)		Height (mm)		GS Median (GSM)		GS Mean		Heterog. (%)		Lipidic (%)	
	R	*p*	R	*p*	R	*p*	R	*p*	R	*p*	R	*p*	R	*p*
Area (mm^2^)	1.00		0.84	**<0.001**	0.66	**<0.001**	−0.03	0.649	−0.01	0.869	0.01	0.877	0.04	0.546
Width (mm)	0.84	**<0.001**	1.00		0.37	**<0.001**	−0.14	**0.060**	−0.13	**0.085**	−0.17	**0.023**	0.10	0.184
Height (mm)	0.66	**<0.001**	0.37	**<0.001**	1.00		0.03	0.707	0.05	0.481	0.27	**<0.001**	0.03	0.687
GS median	−0.03	0.649	−0.14	0.060	0.03	0.707	1.00		0.98	**<0.001**	0.46	**<0.001**	−0.85	**<0.001**
GS mean	−0.01	0.869	−0.13	0.085	0.05	0.481	0.98	**<0.001**	1.00		0.56	**<0.001**	−0.88	**<0.001**
Heterog. (%)	0.01	0.877	−0.17	**0.023**	0.27	**<0.001**	0.46	**<0.001**	0.56	**<0.001**	1.00		−0.40	**<0.001**
Lipidic (%)	0.04	0.546	0.10	0.184	0.03	0.687	−0.85	**<0.001**	−0.88	**<0.001**	−0.40	**<0.001**	1.00	
Fib-Lip. (%)	−0.09	0.215	0.02	0.768	−0.19	**0.008**	−0.50	**<0.001**	−0.45	**<0.001**	−0.45	**<0.001**	0.07	0.374
Fibrous (%)	0.00	0.973	−0.10	0.165	0.06	0.412	0.95	**<0.001**	0.97	**<0.001**	0.55	**<0.001**	−0.88	**<0.001**
GS mean 1mm	−0.04	0.616	−0.18	**0.013**	0.08	0.264	0.95	**<0.001**	0.96	**<0.001**	0.57	**<0.001**	−0.85	**<0.001**
Lipidic 1mm (%)	0.04	0.565	0.11	0.144	0.01	0.905	−0.78	**<0.001**	−0.81	**<0.001**	−0.39	**<0.001**	0.92	**<0.001**
Fib-Lip. 1mm (%)	−0.07	0.347	0.06	0.423	−0.18	**0.013**	−0.41	**<0.001**	−0.37	**<0.001**	−0.46	**<0.001**	−0.03	0.676
Fibrous 1mm (%)	−0.03	0.660	−0.15	**0.036**	0.06	0.447	0.92	**<0.001**	0.94	**<0.001**	0.59	**<0.001**	−0.83	**<0.001**
	Fibrolipidic (%)		Fibrous (%)		GS mean 1 mm		Lipidic 1 mm (%)		Fibrolipidic 1 mm (%)		Fibrous 1 mm (%)			
	R	*p*	R	*p*	R	*p*	R	*p*	R	*p*	R	*p*		
Area (mm^2^)	−0.09	0.215	0.00	0.973	−0.04	0.616	0.04	0.565	−0.07	0.347	−0.03	0.660		
Width (mm)	0.02	0.768	−0.10	0.165	−0.18	**0.013**	0.11	0.144	0.06	0.423	−0.15	**0.036**		
Height (mm)	−0.19	**0.008**	0.06	0.412	0.08	0.264	0.01	0.905	−0.18	**0.013**	0.06	0.447		
GS median	−0.50	**<0.001**	0.95	**<0.001**	0.95	**<0.001**	−0.78	**<0.001**	−0.41	**<0.001**	0.92	**<0.001**		
GS mean	−0.45	**<0.001**	0.97	**<0.001**	0.96	**<0.001**	−0.81	**<0.001**	−0.37	**<0.001**	0.94	**<0.001**		
Heterog, (%)	−0.45	**<0.001**	0.55	**<0.001**	0.57	**<0.001**	−0.39	**<0.001**	−0.46	**<0.001**	0.59	**<0.001**		
Lipidic (%)	0.07	0.374	−0.88	**<0.001**	−0.85	**<0.001**	0.92	**<0.001**	−0.03	0.676	−0.83	**<0.001**		
Fib-Lip. (%)	1.00		−0.54	**<0.001**	−0.47	**<0.001**	0.05	0.531	0.93	**<0.001**	−0.53	**<0.001**		
Fibrous (%)	−0.54	**<0.001**	1.00		0.94	**<0.001**	−0.80	**<0.001**	−0.43	**<0.001**	0.95	**<0.001**		
GS mean 1mm	−0.47	**<0.001**	0.94	**<0.001**	1.00		−0.83	**<0.001**	−0.43	**<0.001**	0.97	**<0.001**		
Lipidic 1mm (%)	0.05	0.531	−0.80	**<0.001**	−0.83	**<0.001**	1.00		−0.03	0.729	−0.82	**<0.001**		
Fib-Lip. 1mm (%)	0.93	**<0.001**	−0.43	**<0.001**	−0.43	**<0.001**	−0.03	0.729	1.00		−0.49	**<0.001**		
Fibrous 1mm (%)	−0.53	**<0.001**	0.95	**<0.001**	0.97	**<0.001**	−0.82	**<0.001**	−0.49	**<0.001**	1.00			

GS: grayscale. Fib-Lip.: Fibrolipidic. 1 mm: first (1st) atherosclerotic plaque superficial millimeter (layer). Heterog.: Heterogeneity.

## Supplementary Materials

The following are available online at https://www.mdpi.com/2308-3425/7/1/11/s1. **Figures:** Figure S1. ROC curve for age as AP presence predictor. Figure S2. ROC curve for BMI as AP presence predictor. Figure S3. ROC curve for HR as AP presence predictor. Figure S4. ROC curve for pSBP as AP presence predictor. Figure S5. ROC curve for pPP as AP presence predictor. Figure S6. ROC curve for cSBP as AP presence predictor. Figure S7. ROC curve for cPP as AP presence predictor. Figure S8. ROC curve for TC as AP presence predictor. Figure S9. ROC curve for glycemia as AP presence predictor. Figure S10. ROC curve for 10-y./FRS-CHD as AP presence predictor. Figure S11. ROC curve for 10-y./FRS-MI as AP presence predictor. Figure S12. ROC curve for 10-y./FRS-Stroke as AP presence predictor. Figure S13. ROC curve for 10-y./FRS-CVD as AP presence predictor. Figure S14. ROC curve for 10-y./FRS-CHD DEATH as AP presence predictor. Figure S15. ROC curve for 10-y./FRS-CVD DEATH as AP presence predictor. Figure S16. ROC curve for 10-y./BNF Risk as AP presence predictor. Figure S17. ROC curve for 10-y./ASSIGN Risk as AP presence predictor. Figure S18. ROC curve for age as AP burden (APB) predictor (Nº of AP: 1 or >1). Figure S19. ROC curve for cPP as APB predictor (Nº of AP: 1 or >1). Figure S20. ROC curve for 10-y./ASSIGN Risk as APB predictor (Nº of AP: 1 or >1). Figure S21. ROC curve for age as APB predictor (Nº of territories: 1 or >1). Figure S22. ROC curve for glycemia as APB predictor (Nº of territories: 1 or >1). Figure S23. ROC curve for 10-y./FRS-Stroke as ABP predictor (Nº of territories, 1 or >1). Figure S24. ROC curve for 10-y./FRS-CVD as APB predictor (Nº of territories: 1 or >1). Figure S25. ROC curve for 10-y./FRS-CHD DEATH as APB predictor (Nº of territories: 1 or >1). Figure S26. ROC curve for 10-y./FRS-CVD DEATH as APB predictor (Nº of territories, 1 or >1). Figure S27. ROC curve for 10-y./BNF as APB predictor (Nº of territories: 1 or >1). Figure S28. ROC curve for 10-y./ASSIGN as APB predictor (Nº of territories:1 or >1). Figure S29. ROC curve for age as AP surface area predictor [<p75th or ≥p75th]. Figure S30. ROC curve for pSBP as AP surface area predictor [<p75th or ≥p75th]. Figure S31. ROC curve for pPP as AP surface area predictor [<p75th or ≥p75th]. Figure S32. ROC curve for cSBP as AP surface area predictor [<p75th or ≥p75th]. Figure S33. ROC curve for cPP as AP surface area predictor [<p75th or ≥p75th]. Figure S34. ROC curve for TC/HDL as AP surface area predictor [<p75th or ≥p75th]. Figure S35. ROC curve for LDL/HDL as AP surface area predictor [<p75th or ≥p75th]. Figure S36. ROC curve for 10-y./FRS-CHD as AP surface area predictor [<p75th or ≥p75th]. Figure S37. ROC curve for 10-y./FRS-MI as AP surface area predictor [<p75th or ≥p75th]. Figure S38. ROC curve for 10-y./FRS-Stroke as AP surface area predictor [<p75th or ≥p75th]. Figure S39. ROC curve for 10-y./FRS-CVD as AP surface area predictor [<p75th or ≥p75th]. Figure S40. ROC curve for 10-y./FRS-CHD DEATH as AP surface area predictor [<p75th or ≥p75th]. Figure S41. ROC curve for 10-y./FRS-CVD DEATH as AP surface area predictor [<p75th or ≥p75th]. Figure S42. ROC curve for 10-y./BNF as AP surface area predictor [<p75th or ≥p75th]. Figure S43. ROC curve for 10-y./ASSIGN as AP surface area predictor [<p75th or ≥p75th]. Figure S44. ROC curve for TC/HDL as AP composition predictor (0: fibrous, 1: fibrolipidic). Figure S45. ROC curve for LDL/HDL as AP composition predictor (0: fibrous, 1: fibrolipidic). Figure S46. ROC curve for LogTG/HDL as AP composition predictor (0: fibrous, 1: fibrolipidic). Figure S47. ROC curve for TC/HDL as AP composition predictor [GSM ≤105 or >105]. Figure S48. ROC curve for LDL/HDL as AP composition predictor [GSM ≤105 or >105]. Figure S49. ROC curve for LogTG/HDL as AP composition predictor [GSM ≤105 or >105]. Figure S50. ROC curve for TC/HDL as AP composition predictor [grayscale media ≤110 or >110]. Figure S51. ROC curve for LDL/HDL as AP composition predictor [GS Media ≤110 or >110]. Figure S52. ROC curve for LogTG/HDL as AP composition predictor [GS Media ≤110 or >110]. Figure S53. ROC curve for TC/HDL as AP composition predictor [GS Media 1st mm ≤110 or >110]. Figure S54. ROC curve for LDL/HDL as AP composition predictor [GS Media 1st mm ≤110 or >110]. Figure S55. ROC curve for LogTG/HDL as AP composition predictor [GS Media 1st mm ≤110 or >110]. Figure S56. Comparison of ROC curves for age and BMI as AP presence predictors. Figure S57. Comparison of ROC curves for age and HR as AP presence predictors. Figure S58. Comparison of ROC curves for age and pSBP as AP presence predictors. Figure S59. Comparison of ROC curves for age and pPP as AP presence predictors. Figure S60. Comparison of ROC curves for age and cSBP as AP presence predictors. Figure S61. Comparison of ROC curves for age and cPP as AP presence predictors. Figure S62. Comparison of ROC curves for age and TC as AP presence predictors. Figure S63. Comparison of ROC curves for age and glycemia as AP presence predictors. Figure S64. Comparison of ROC curves for age and 10-y./FRS-CHD as AP presence predictors. Figure S65. Comparison of ROC curves for age and 10-y./FRS-MI as AP presence predictors. Figure S66. Comparison of ROC curves for age and 10-y./FRS-Stroke as AP presence predictors. Figure S67. Comparison of ROC curves for age and 10-y./FRS-CVD as AP presence predictors. Figure S68. Comparison of ROC curves for age and 10-y./BNF as AP presence predictors. Figure S69. Comparison of ROC curves for BMI and 10-y./FRS-CHD as AP presence predictors. Figure S70. Comparison of ROC curves for BMI and 10-y./FRS-MI as AP presence predictors. Figure S71. Comparison of ROC curves for BMI and 10-y./FRS-Stroke as AP presence predictors. Figure S72. Comparison of ROC curves for BMI and 10-y./FRS-CVD as AP presence predictors. Figure S73. Comparison of ROC curves for BMI and 10-y./FRS-CHD DEATH as AP presence predictors. Figure S74. Comparison of ROC curves for BMI and 10-y./FRS-CVD DEATH as AP presence predictors. Figure S75. Comparison of ROC curves for BMI and 10-y./BNF as AP presence predictors. Figure S76. Comparison of ROC curves for BMI and 10-y./ASSIGN as AP presence predictors. Figure S77. Comparison of ROC curves for HR and 10-y./FRS-CHD as AP presence predictors. Figure S78. Comparison of ROC curves for HR and 10-y./FRS-Stroke as AP presence predictors. Figure S79. Comparison of ROC curves for HR and 10-y./FRS-CVD as AP presence predictors. Figure S80. Comparison of ROC curves for HR and 10-y./FRS-CHD DEATH as AP presence predictors. Figure S81. Comparison of ROC curves for HR and 10-y./FRS-CVD DEATH as AP presence predictors. Figure S82. Comparison of ROC curves for HR and 10-y./BNF as AP presence predictors. Figure S83. Comparison of ROC curves for HR and 10-y./ASSIGN as AP presence predictors. Figure S84. Comparison of ROC curves for pSBP and cSBP as AP presence predictors. Figure S85. Comparison of ROC curves for pSBP and 10-y./FRS-CHD as AP presence predictors. Figure S86. Comparison of ROC curves for pSBP and 10-y./FRS-MI as AP presence predictors. Figure S87. Comparison of ROC curves for pSBP and 10-y./FRS-Stroke as AP presence predictors. Figure S88. Comparison of ROC curves for pSBP and 10-y./FRS-CVD as AP presence predictors. Figure S89. Comparison of ROC curves for pSBP and 10-y./FRS-CHD DEATH as AP presence predictors. Figure S90. Comparison of ROC curves for pSBP and 10-y./FRS-CVDDEATH as AP presence predictors. Figure S91. Comparison of ROC curves for pSBP and 10-y./BNF as AP presence predictors. Figure S92. Comparison of ROC curves for pSBP and 10-y./ASSIGN as AP presence predictors. Figure S93. Comparison of ROC curves for pPP and cSBP as AP presence predictors. Figure S94. Comparison of ROC curves for pPP and cPP as AP presence predictors [yes/no]. Figure S95. Comparison of ROC curves for pPP and 10-y./FRS-CHD as AP presence predictors. Figure S96. Comparison of ROC curves for pPP and 10-y./FRS-MI as AP presence predictors. Figure S97. Comparison of ROC curves for pPP and 10-y./FRS-Stroke as AP presence predictors. Figure S98. Comparison of ROC curves for pPP and 10-y./FRS-CVD as AP presence predictors. Figure S99. Comparison of ROC curves for pPP and 10-y./FRS-CHD DEATH as AP presence predictors. Figure S100. Comparison of ROC curves for pPP and 10-y./FRS-CVD DEATH as AP presence predictors. Figure S101. Comparison of ROC curves for pPP and 10-y./BNF as AP presence predictors. Figure S102. Comparison of ROC curves for pPP and 10-y./ASSIGN as AP presence predictors. Figure S103. Comparison of ROC curves for cSBP and 10-y./FRS-CHD as AP presence predictors. Figure S104. Comparison of ROC curves for cSBP and 10-y./FRS-MI as AP presence predictors. Figure S105. Comparison of ROC curves for cSBP and 10-y./FRS-Stroke as AP presence predictors. Figure S106. Comparison of ROC curves for cSBP and 10-y./FRS-CVD as AP presence predictors. Figure S107. Comparison of ROC curves for cSBP and 10-y./CVD-CHD as AP presence predictors. Figure S108. Comparison of ROC curves for cSBP and 10-y./FRS-CVD DEATH as AP presence of AP predictors. Figure S109. Comparison of ROC curves for cSBP and 10-y./BNF as AP presence predictors. Figure S110. Comparison of ROC curves for cSBP and 10-y./ASSIGN as AP presence predictors. Figure S111. Comparison of ROC curves for cPP and 10-y./FRS-CHD as AP presence predictors. Figure S112. Comparison of ROC curves for cPP and 10-y./FRS-Stroke as AP presence predictors. Figure S113. Comparison of ROC curves for cPP and 10-y./FRS-CVD as AP presence predictors. Figure S114. Comparison of ROC curves for cPP and 10-y./FRS-CHD DEATH as AP presence predictors. Figure S115. Comparison of ROC curves for cPP and 10-y./FRS-CVD DEATH as AP presence predictors. Figure S116. Comparison of ROC curves for cPP and 10-y./BNF as AP presence predictors. Figure S117. Comparison of ROC curves for cPP and 10-y./ASSIGN as AP presence predictors. Figure S118. Comparison of ROC curves for TC and 10-y./FRS-CHD as AP presence predictors. Figure S119. Comparison of ROC curves for TC and 10-y./FRS-Stroke as AP presence predictors. Figure S120. Comparison of ROC curves for TC and 10-y./FRS-CVD as AP presence predictors. Figure S121. Comparison of ROC curves for TC and 10-y./FRS-CHD as AP presence predictors. Figure S122. Comparison of ROC curves for TC and 10-y./FRS-CVD as AP presence predictors. Figure S123. Comparison of ROC curves for TC and 10-y./BNF as AP presence predictors. Figure S124. Comparison of ROC curves for TC and 10-y./ASSIGN as AP presence predictors. Figure S125. Comparison of ROC curves for glycemia and 10-y./FRS-CHD as AP presence predictors. Figure S126. Comparison of ROC curves for glycemia and 10-y./FRS-Stroke as AP presence predictors. Figure S127. Comparison of ROC curves for glycemia and 10-y./FRS-CVD as AP presence predictors. Figure S128. Comparison of ROC curves for glycemia and 10-y./FRS-CHD DEATH as AP presence predictors. Figure S129. Comparison of ROC curves for glycemia and 10-y./FRS-CVD DEATH as AP presence predictors. Figure S130. Comparison of ROC curves for glycemia and 10-y./BNF as AP presence predictors. Figure S131. Comparison of ROC curves for glycemia and 10-y./ASSIGN as AP presence predictors. Figure S132. Comparison of ROC curves for 10-y./FRS-CHD and 10-y./FRS-MI as AP presence predictors. Figure S133. Comparison of ROC curves for 10-y./FRS-CHD and 10-y./FRS-Stroke as AP presence predictors. Figure S134. Comparison of ROC curves for 10-y./FRS-CHD and 10-y./FRS-CVD as AP presence predictors. Figure S135. Comparison of ROC curves for 10-y./FRS-CHD and 10-y./FRS-CHD DEATH as AP presence predictors Figure S136. Comparison of ROC curves for 10-y./FRS-CHD and 10-y./FRS-CVD DEATH as AP presence predictors. Figure S137. Comparison of ROC curves for 10-y./FRS-CHD and 10-y./BNF as AP presence predictors. Figure S138. Comparison of ROC curves for 10-y./FRS-CHD and 10-y./ASSIGN as AP presence predictors. Figure S139. Comparison of ROC curves for 10-y./FRS-MI and 10-y./FRS-Stroke as AP presence predictors. Figure S140. Comparison of ROC curves for 10-y./FRS-MI and 10-y./FRS-CVD as AP presence predictors. Figure S141. Comparison of ROC curves for 10-y./FRS-MI and 10-y./FRS-CHD DEATH as AP presence predictors. Figure S142. Comparison of ROC curves for 10-y./FRS-MI and 10-y./FRS-CVD DEATH as AP presence predictors. Figure S143. Comparison of ROC curves for 10-y./FRS-MI and 10-y./BNF as AP presence predictors. Figure S144. Comparison of ROC curves for 10-y./FRS-MI and 10-y./ASSIGN as AP presence predictors. Figure S145. Comparison of ROC curves for 10-y./FRS-Stroke and 10-y./ASSIGN as AP presence predictors. Figure S146. Comparison of ROC curves for 10-y./FRS-CVD and 10-y./FRS-CVD DEATH as AP presence predictors. Figure S147. Comparison of ROC curves for 10-y./FRS-CVD and 10-y./BNF as AP presence predictors. Figure S148. Comparison of ROC curves for 10-y./FRS-CVD and 10-y./ASSIGN as AP presence predictors. Figure S149. Comparison of ROC curves for 10-y./FRS-CHD DEATH and 10-y./FRS-CVD DEATH as AP presence predictors. Figure S150. Comparison of ROC curves for 10-y./FRS-CHD DEATH and 10-y./BNF as AP presence predictors. Figure S150. Comparison of ROC curves for 10-y./FRS-CHD DEATH and 10-y./BNF as AP presence predictors. Figure S151. Comparison of ROC curves for 10-y./FRS-CHD DEATH and 10-y./ASSIGN as AP presence predictors. Figure S152. Comparison of ROC curves for 10-y./FRS-CVD DEATH and 10-y./BNF as AP presence predictors. Figure S153. Comparison of ROC curves for 10-y./FRS-CVD DEATH and 10-y./ASSING as AP presence predictors. Figure S154. Comparison of ROC curves for 10-y./BNF and 10-y./ASSING as AP presence predictors. Figure S155. Comparison of ROC curves for 10-y./FRS-MI and 10-y./FRS-CHD DEATH as AP presence predictors. Figure S156. Comparison of ROC curves for 10-y./FRS-MI and 10-y./BNF as AP presence predictors. **Tables:** Table S1. Comparison of geometric AP properties association with lipid ratios or 10-y./CVR levels (William’s Test). Table S2. Comparison of global composition AP properties association with lipid ratios or 10-y./CVR levels (William’s Test). Table S3. Comparison of 1st mm composition AP properties association with lipid ratios or 10-y./CVR levels (William’s Test). Table S4. Association between AP presence or burden and CRFs (Logistic regression analysis). Table S5. Association between AP geometric properties and CRFs (Logistic regression analysis). Table S6. Prediction of AP presence (Criterion values and coordinates of the ROC curve): age [y.] as variable test. Table S7. Prediction of AP presence (Criterion values and coordinates of the ROC curve): BMI [Kg./m^2^] as variable test. Table S8. Prediction of AP presence(Criterion values and coordinates of the ROC curve): HR [b.p.m.] as variable test. Table S9. Prediction of AP presence (Criterion values and coordinates of the ROC curve): pSBP [mmHg] as variable test. Table S10. Prediction of AP presence (Criterion values and coordinates of the ROC curve): pPP [mmHg] as variable test. Table S11. Prediction of AP presence (Criterion values and coordinates of the ROC curve): cSBP [mmHg] as variable test. Table S12. Prediction of AP presence (Criterion values and coordinates of the ROC curve): cPP [mmHg] as variable test. Table S13. Prediction of AP presence Criterion values and coordinates of the ROC curve): TC[mg/dL] as variable test. Table S14. Prediction of AP presence (Criterion values and coordinates of the ROC curve): Glycemia [mg/dL] as variable test. Table S15. Prediction of AP presence (Criterion values and coordinates of the ROC curve): 10-y./FRS-CHD [%] as variable test. Table S16. Prediction of AP presence (Criterion values and coordinates of the ROC curve): 10-y./FRS-MI [%] as variable test. Table S17. Prediction of AP presence (Criterion values and coordinates of the ROC curve): 10-y./FRS-Stroke [%] as variable test. Table S18. Prediction of AP presence (Criterion values and coordinates of the ROC curve): 10-y./FRS-CVD [%] as variable test. Table S19. Prediction of AP presence (Criterion values and coordinates of the ROC curve): 10-y./FRS-CHD DEATH [%] as variable test. Table S20. Prediction of AP presence (Criterion values and coordinates of the ROC curve): 10-y./FRS-CVD DEATH [%] as variable test. Table S21. Prediction of AP presence (Criterion values and coordinates of the ROC curve): 10-y./BNF Risk Score [%] as variable test. Table S22. Prediction of AP presence (Criterion values and coordinates of the ROC curve): 10-y./ASSIGN Risk Score [%] as variable test. Table S23. Prediction of APB [Nº of AP, 1 or >1] (Criterion values and coordinates of the ROC curve): age [y.] as variable test. Table S24. Prediction of APB [Nº of AP, 1 or >1] (Criterion values and coordinates of the ROC curve): cPP [mmHg] as variable test. Table S25. Prediction of APB [Nº of AP, 1 or >1] (Criterion values and coordinates of the ROC curve): 10-y./ASSIGN Risk Score [%] as variable test. Table S26. Prediction of APB [Nº of territories, 1 or >1] (Criterion values and coordinates of the ROC curve): age [y.] as variable test. Table S27. Prediction of APB [Nº of territories, 1 or >1] (Criterion values and coordinates of the ROC curve): glycemia [mg/dL] as variable test. Table S28. Prediction of APB [Nº of territories, 1 or >1] (Criterion values and coordinates of the ROC curve): 10-y./FRS-Stroke [%] as variable test. Table S29. Prediction of APB [Nº of territories, 1 or >1] (Criterion values and coordinates of the ROC curve): 10-y./FRS-CVD [%] as variable test. Table S30. Prediction of APB [Nº of territories, 1 or >1] (Criterion values and coordinates of the ROC curve): 10-y./FRS-CHD DEATH [%] as variable test. Table S31. Prediction of APB [Nº of territories, 1 or >1] (Criterion values and coordinates of the ROC curve): 10-y./FRS-CVD DEATH [%] as variable test. Table S32. Prediction of APB [Nº of territories, 1 or >1] (Criterion values and coordinates of the ROC curve): 10-y./BNF Risk Score [%] as variable test. Table S33. Prediction of APB [Nº of territories, 1 or >1] (Criterion values and coordinates of the ROC curve): 10-y./ASSIGN Risk Score [%] as variable test. Table S34. Prediction of AP surface area [<p75th or ≥p75th] (Criterion values and coordinates of the ROC curve): age [y.] as variable test. Table S35. Prediction of AP surface area [<p75th or ≥p75th] (Criterion values and coordinates of the ROC curve): pSBP [mmHg] as variable test. Table S36. Prediction of AP surface area [<p75th or ≥p75th] (Criterion values and coordinates of the ROC curve): pPP [mmHg] as variable test. Table S37. Prediction of AP surface area [<p75th or ≥p75th] (Criterion values and coordinates of the ROC curve): cSBP [mmHg] as variable test. Table S38. Prediction of AP surface area [<p75th or ≥p75th] (Criterion values and coordinates of the ROC curve): central PP [mmHg] as variable test. Table S39. Prediction of AP surface area [<p75th or ≥p75th] (Criterion values and coordinates of the ROC curve): TC/HDL as variable test. Table S40. Prediction of AP surface area [<p75th or ≥p75th] (Criterion values and coordinates of the ROC curve): LDL/HDL as variable test. Table S41. Prediction of AP surface area [<p75th or ≥p75th] (Criterion values and coordinates of the ROC curve): 10-y./FRS-CHD [%] as variable test. Table S42. Prediction of AP surface area [<p75th or ≥p75th] (Criterion values and coordinates of the ROC curve): 10-y./FRS-MI [%] as variable test. Table S43. Prediction of AP surface area [<p75th or ≥p75th] (Criterion values and coordinates of the ROC curve): 10-y./FRS-Stroke [%] as variable test. Table S44. Prediction of AP surface area [<p75th or ≥p75th] (Criterion values and coordinates of the ROC curve): 10-y./FRS-CVD [%] as variable test. Table S45. Prediction of AP surface area [<p75th or ≥p75th] (Criterion values and coordinates of the ROC curve): 10-y./FRS-CHD DEATHC [%] as variable test. Table S46. Prediction of AP surface area [<p75th or ≥p75th] (Criterion values and coordinates of the ROC curve): 10-y./FRS-CVD DEATH [%] as variable test. Table S47. Prediction of AP surface area [<p75th or ≥p75th] (Criterion values and coordinates of the ROC curve): 10-y./BNF Risk Score [%] as variable test. Table S48. Prediction of AP surface area [<p75th or ≥p75th] (Criterion values and coordinates of the ROC curve): 10-y./ASSIGN Risk Score [%] as variable test. Table S49. Prediction of AP composition [0:fibrous, 1:fibrolipidic] (Criterion values and coordinates of the ROC curve): TC/HDL as variable test. Table S50. Prediction of AP composition [0:fibrous, 1:fibrolipidic] (Criterion values and coordinates of the ROC curve): LDL/HDL as variable test. Table S51. Prediction of AP composition [0:fibrous, 1:fibrolipidic] (Criterion values and coordinates of the ROC curve): LogTG/HDL as variable test. Table S52. Prediction of AP composition [GSM ≤105 or >105] (Criterion values and coordinates of the ROC curve): TC/HDL as variable test. Table S53. Prediction of AP composition [GSM ≤105 or >105] (Criterion values and coordinates of the ROC curve): LDL/HDL as variable test. Table S54. Prediction of AP composition [GSM ≤105 or >105] (Criterion values and coordinates of the ROC curve): LogTG/HDL as variable test. Table S55. Prediction of AP composition [GS Media ≤105 or >105] (Criterion values and coordinates of the ROC curve): TC/HDL as variable test. Table S56. Prediction of AP composition [GS Media ≤110 or >110] (Criterion values and coordinates of the ROC curve): LDL/HDL as variable test. Table S57. Prediction of AP composition [GS Media ≤110 or >110] (Criterion values and coordinates of the ROC curve): LogTG/HDL as variable test. Table S58. Prediction of AP composition [GS Media 1st mm ≤110 or >110] (Criterion values and coordinates of the ROC curve): TC/HDL as variable. Table S59. Prediction of AP composition [GS Media 1st mm ≤110 or >110] (Criterion values and coordinates of the ROC curve): LDL/HDL as variable test. Table S60. Prediction of AP composition [GS Media 1st mm ≤110 or >110] (Criterion values and coordinates of the ROC curve): LogTG/HDL ratio as variable test. Table S61. Comparison of ROC curves for AP presence prediction. Table S62. Comparison of ROC curves for APB prediction (Nº of AP, 1 or >1). Table S63. Comparison of ROC curves for APB (Nº of territories, 1 or >1) prediction. Table S64. Comparison of ROC curves for surface area (<p75 or ≥p75) prediction. Table S65. Comparison between association levels obtained between AP geometric and composition properties (William’s Test).

## Abbreviations

+LR: -LR	Positive and negative likelihood ratio: respectively
AI	Atherogenic Index
ANCOVA	Analysis of Covariance
ASSIGN	Scottish Intercollegiate Guidelines Network (SIGN), Cardiovascular Risk Score
AUC	Area Under Curve or Area under ROC Curve
BMI	Body Mass Index
BNF	British National Formulary Cardiovascular Risk Score
BP	Blood Pressure
CCA	Common Carotid Artery
CFA	Common Femoral Artery
CHD	Coronary Heart Disease
CRFs	Cardiovascular Risk Factors
CUiiDARTE	Centro Universitario de Investigación, Innovación y Diagnóstico Arterial
CV	Cardiovascular
CVD	Cardiovascular Disease
CVR	Cardiovascular risk
FRS	Framingham Risk Score
GSM	Grayscale Median
HDL	High-density Lipoprotein Cholesterol
HR	Heart Rate
HT	Arterial Hypertension
IMT	Intima-media Thickness
LDL	Low-density Lipoprotein Cholesterol
LogRM	Logistic Regression Models
MI	Myocardial Infarction
PAI	Plasma Atherogenic Index
pBP, cBP	Peripheral (Brachial) and Central (Aortic) Blood Pressure, respectively.
pDBP, cDBP	Peripheral (Brachial) and Central (Aortic) Diastolic Blood Pressure, respectively.
pMBP	Peripheral (Brachial) Mean Blood Pressure
pPP, cPP	Peripheral (Brachial) and Central (Aortic) Pulse Pressure, respectively.
pSBP, cSBP	Peripheral (Brachial) and Central (Aortic) Systolic Blood Pressure, respectively.
R	Correlation Coefficient
ROC	Receiver Operating Characteristic Curve
SE	Sensibility
SP	Specificity
TC	Total Cholesterol
TG	Triglycerides
